# *Leishmania donovani* parasite requires Atg8 protein for infectivity and survival under stress

**DOI:** 10.1038/s41419-019-2038-7

**Published:** 2019-10-24

**Authors:** Sagnik Giri, Chandrima Shaha

**Affiliations:** Cell Death and Differentiation Laboratory, National Institute of Immunology, New Delhi, 110067 India

**Keywords:** Macroautophagy, Macroautophagy

## Abstract

The importance of autophagy in parasites with a digenetic life cycle like *Leishmania* spp. is significant. The parasite survives as promastigotes in the insect gut and as immotile amastigotes in mammals. This study demonstrates increased autophagy in *Leishmania* parasite during progression of in vitro life cycle and upon exposure to stress stimuli like starvation, oxidative stress, and drugs. Autophagy inhibition during stress exposure increased cell death, indicating the importance of autophagy in cellular defense against adverse conditions. Atg8 protein, a homolog of mammalian autophagy protein LC3 is expressed in *Leishmania* parasite but its function remains unknown. Overexpression of Atg8 (*Atg8-*OE) rendered the parasites resistant to stress and capable of infecting macrophages in substantial numbers; however, disruption of the *Atg8* gene (Δ*Atg8*) resulting in suppression of Atg8 protein expression, increased susceptibility to stress and reduced the capability to cause infection. A critical event in the *Leishmania* parasite lifecycle is the differentiation of promastigote forms to the disease causing amastigote forms. The failure of Δ*Atg8* parasites lacking Atg8 protein to differentiate into amastigotes, unlike the *Atg8-*OE and vector-transfected parasites, clearly indicated Atg8 involvement in a crucial event. The inability of Δ*Atg8* parasites to infect macrophages in vitro was verified in an in vivo mouse model of leishmaniases where infection could not be induced by the Δ*Atg8* parasites. Autophagy is known to be involved in the remodeling of damaged organelles. The accumulation of Atg8 around damaged mitochondria suggested increase of autophagy in the vicinity of the organelle. This buildup was prevented when mitochondria generated reactive oxygen species that were quenched, suggesting them as possible signaling molecules for sensing mitochondrial instability. In summary, our study provides new evidences for a crucial role of Atg8 protein in sustaining *Leishmania* parasite survival during life cycle and stress exposure, differentiation to amastigotes, and their infective abilities.

## Introduction

Autophagy is an intracellular degradation system that delivers cellular constituents including cytosolic proteins and damaged organelles to the lysosome for digestion by enzymes^1,2^. Analysis of autophagic processes known to help remodeling of cells has attracted considerable attention because of the influence of autophagy in different cellular functions^1,2^. For organisms where change in cell shape and size is essential during the life cycle, autophagy plays a very important role^3^. One such organism, the *Leishmania* parasite, infects mammals and causes a group of diseases collectively called leishmaniases^4,5^. Three forms of the disease exist, the potentially fatal systemic visceral form, caused primarily by *Leishmania donovani* and *Leishmania infantum;* the cutaneous and the mucocutaneous disease forms caused by *Leishmania major, Leishmania tropica,* and the related parasites of the same genus. These parasites have a digenetic life cycle where the free-swimming procyclic promastigote form undergoes differentiation to enter into an infective metacyclic stage, and finally after infection, differentiates into the disease causing rounded amastigote forms that live within the macrophages. The occurrence of macroautophagy in *L. major* and the involvement of several Atg or autophagy-related proteins have been elegantly shown in several studies^6,7^. These Atg proteins are intimately associated with the regulation of macroautophagy (henceforth referred to as autophagy), and the requirement of a functional Atg12–Atg5 conjugation system for Atg8-dependent autophagy in *L. major* has been demonstrated^6–9^. However, the consequences of the absence of Atg8 protein on the formation of autophagosomes, response to drugs, and infectivity are not known.

The presence of Atg8 in *Leishmania* parasites was shown in prior studies where Atg8 conjugation to phosphatidylethanolamine (PE) to form membrane-bound Atg8 was demonstrated^7,9^. In the later stages of autophagosome formation, Atg8 is cleaved by Atg4 to form membrane-bound PE-conjugated Atg8 (Atg8-II), which localizes to the pre-autophagosomes and facilitates fusion between autophagosome and the lysosome^7,9,10^. The importance of parasite autophagy was first described in studies where overexpression of VPS4-defective mutant, a dominant-negative ATPase involved in disassembly of endosome-sorting complexes for transport of multivesicular bodies, inhibited parasite differentiation to the obligate infective metacyclic form, thereby affecting virulence^6^. This finding was indicative of the requirement of late endosome or autophagic function for differentiation to the metacyclic form. Consequently, well-designed studies from Williams et al. showed the presence of four subfamilies of *Atg8* genes in *L. major*, and the Atg8 protein was shown to be instrumental in autophagosome formation during starvation and differentiation^9^. In contrast to *L. major, L. donovani* expresses two copies of *Atg8* gene on chromosome 19 as identified from NCBI nucleotide database (https://www.ncbi.nlm.nih.gov/nucleotide/); one of them expresses full-length Atg8 protein. Later studies in *L. major* revealed a functional Atg5–Atg12 conjugation system that prompts Atg8-dependent autophagosome formation associated with the mitochondrion under nutrient stress^11^. Mutation of *Atg5* led to mitochondrial abnormality^11^, suggesting a possible link between Atg proteins and mitochondrial health.

The idea of autophagy possibly playing a vital role in *Leishmania* parasite survival prompted us to explore the functional role of the Atg8 protein in *L. donovani*. We demonstrate that inhibition of autophagy by a chemical inhibitor or the absence of Atg8 protein (Δ*Atg8*) makes the parasites more vulnerable to unfavorable conditions created by oxidative stress, while overexpression of Atg8 (*Atg8-*OE) makes them resistant to such insults. Importantly, the absence of Atg8 protein represses the differentiation of promastigotes to amastigotes and inhibits infection of macrophages in vitro. In an in vivo mouse model of leishmaniasis, Δ*Atg8* parasites were unable to cause substantial infection. Under mitochondrial but not genotoxic stress in vitro, the Atg8 protein migrated to the vicinity of the damaged mitochondria, suggesting an association between mitochondrial dysfunction and translocation of the Atg8-positive autophagosomes. This migration and accumulation of Atg8 protein was reduced when mitochondria-generated reactive oxygen species (ROS) were quenched. Overall, our findings suggest a crucial role of Atg8 protein in the survival of the *Leishmania* parasite during life cycle and stress situations, differentiation to amastigotes, and competency to infect.

## Results

### Autophagy regulated by *Atg8* is essential during the in vitro life cycle of *Leishmania* parasite

**Fig. 1:**
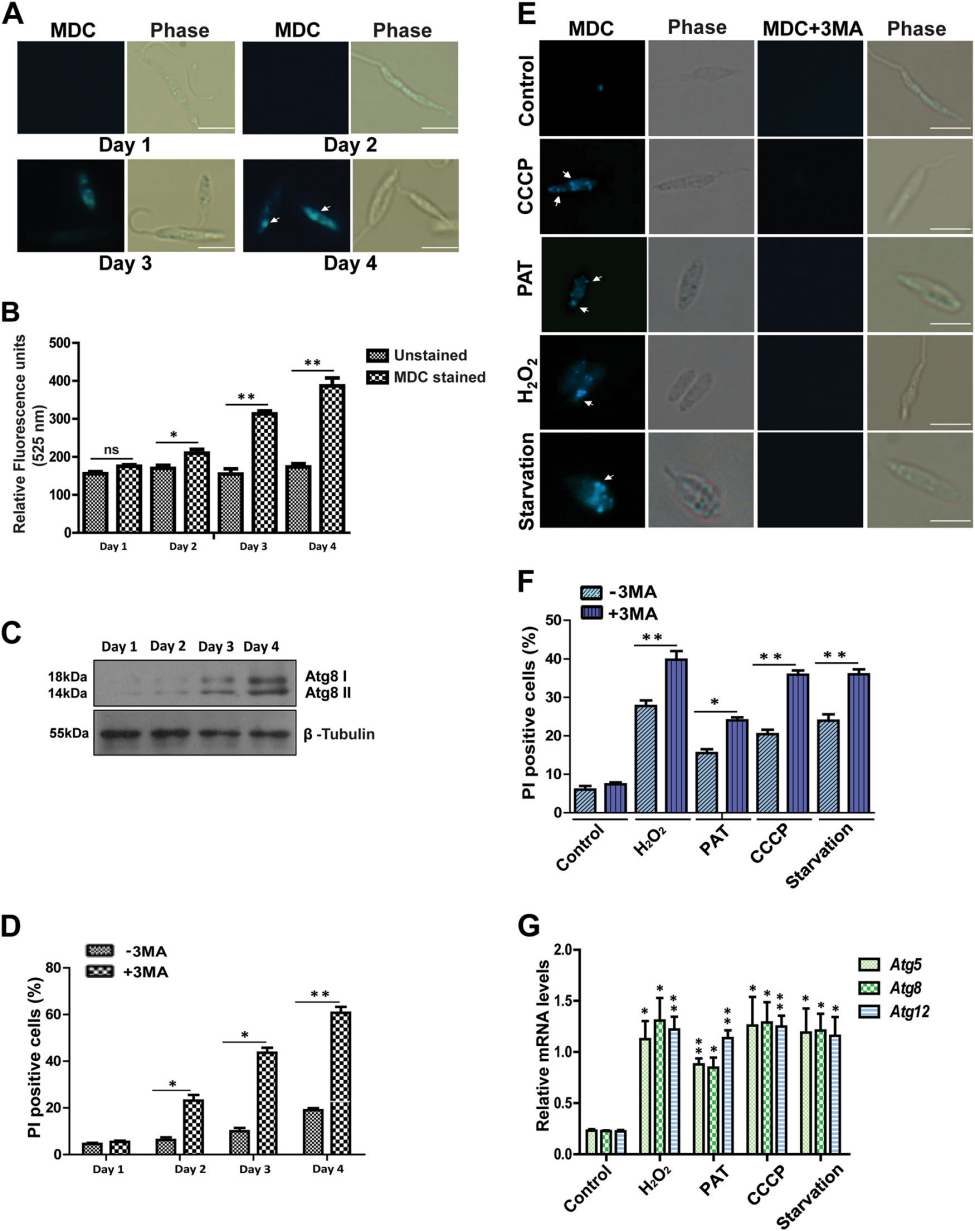
Inhibition of autophagy leads to cell death. **a** Autophagy levels of wild-type parasites were assessed post culture at a starting concentration of 10^6^ cells ml^−1^ for the indicated number of days by staining the autophagic vacuoles with MDC (10 µM). Progressive increase in the number of vacuoles was observed as parasites entered the late log phase in culture. Scale bars represent 5 μm. **b** MDC staining was quantified by fluorometry and represented by the bar graph. Data represent mean ± SEM (*n* = 3). **c** Western blot analysis was carried out to ascertain the day wise change in native Atg8 levels in wild-type parasites. β-tubulin was used as control. Note the increase in Atg8 protein with time. **d** The effect of autophagy inhibition by 3-MA (5 mM) on parasite viability was assessed under normal culture conditions. In total, 10^6^ parasites were sampled out from growing culture with or without 3-MA everyday (days 1–4). Note that blocking autophagy with 3-MA resulted in significant death of the parasites. Data represent mean ± SEM (*n* = 3), *P < 0.05, **P < 0.005. **e** Parasites were treated with H_2_O_2_ (100 µM), PAT (250 µM), and CCCP (200 µM) for 6 h and stained with MDC. Starved parasites deprived of FCS were used as positive controls for autophagy induction. A separate set was treated with 3-MA to inhibit autophagy. Note significant MDC staining in treated and starved groups as compared with VT cells. Scale bars represent 5 μm. **f** Cell death assay after drug treatments analyzed by PI staining shows increased cell death when autophagy was blocked. Data represent mean ± SEM (*n* = 3), NS, nonsignificant. **g** RT-qPCR was carried out to check the RNA levels of the major autophagic genes: *Atg5*, *Atg8*, and *Atg12* upon exposure to the drugs. Note that *Atg5*, *Atg8*, and *Atg12* gene expression increased as compared with *Ld* actin control. Data represent mean ± SEM (*n* = 3), *P < 0.01, **P *<* 0.001

Differentiation of *Leishmania* parasites involves critical regulation of cellular processes for adjusting to adverse conditions in different hosts involving changes in shape and size and adapting to different temperatures and pH^12,13^. Therefore, housekeeping activities required for cellular homeostasis like autophagy are expected to play an important role during the parasite life cycle. To understand the role of autophagic processes in the survival and infective abilities of the parasite, we utilized an in vitro life cycle system frequently used for studying the biology of trypanosomatid parasites^14^. The parasites transit through a log phase to the stationary phase during their life cycle in vitro with considerable changes in biochemistry, shape, and size, and therefore this was an ideal culture system to trace the autophagic changes^6^. Autophagy was observed during in vitro life cycle progression by staining with monodansylcadaverine (MDC), a fluorescent compound that gets incorporated into multilamellar bodies during autophagosome formation, and is used as a probe for detection of autophagic vacuoles. Microscopic observations showed an increase in MDC staining on day 4 of the in vitro life cycle (Fig. 1a)^15^. Estimation of MDC staining by using fluorometry corroborates the evidence for MDC staining enhancement on day 4 (Fig. 1b). Further confirmation of enhancement of autophagy is shown by an increase in Atg8-II expression levels on western blots of extracts derived from parasites at different days of culture (Fig. 1c). Therefore, multiple methods of detection indicated an increase in autophagy on day 4 of the in vitro life cycle. This brought us to the question of the essential nature of autophagy for survival; therefore, we designed experiments where viability was determined after autophagy was blocked by using 3-methyladenine (3-MA), capable of repressing autophagy through inhibition of type I and type III phosphatidylinositol-3-kinases (PI3K). Blocking of autophagy resulted in low parasite viability (Fig. 1d), suggesting a link between reduction of autophagy and cell survival. Autophagic activity is known to come into play during maintenance of cellular homeostasis under stress^16–18^. Therefore, we used several stress inducers like H_2_O_2_, which spontaneously generates hydroxyl ions in physiological conditions^19^, carbonyl cyanide m-chlorophenylhydrazone (CCCP)^20^, a respiratory chain uncoupler that generates ROS, and potassium antimonyl tartrate (PAT), a drug used against *Leishmania* parasite that acts through generation of ROS^21^. As a positive control, another group of parasites were subjected to starvation to mimic a condition where mammalian cells initiate autophagic activities^22–24^. A significant increase in the number of autophagic vacuoles was seen in the parasites subjected to all three stress conditions and during starvation (Fig. 1e). Treatment with 3-MA during exposure to stress, caused a significant reduction in the number of the MDC-stained vacuoles in all groups (Fig. 1e) accompanied by a significant reduction in survival (Fig. 1f). This suggested a necessary role of autophagy in cellular protection during stress. Overall, this part of the data confirmed augmented autophagy during progression of the life cycle in vitro as well as during stress exposure.

**Fig. 2:**
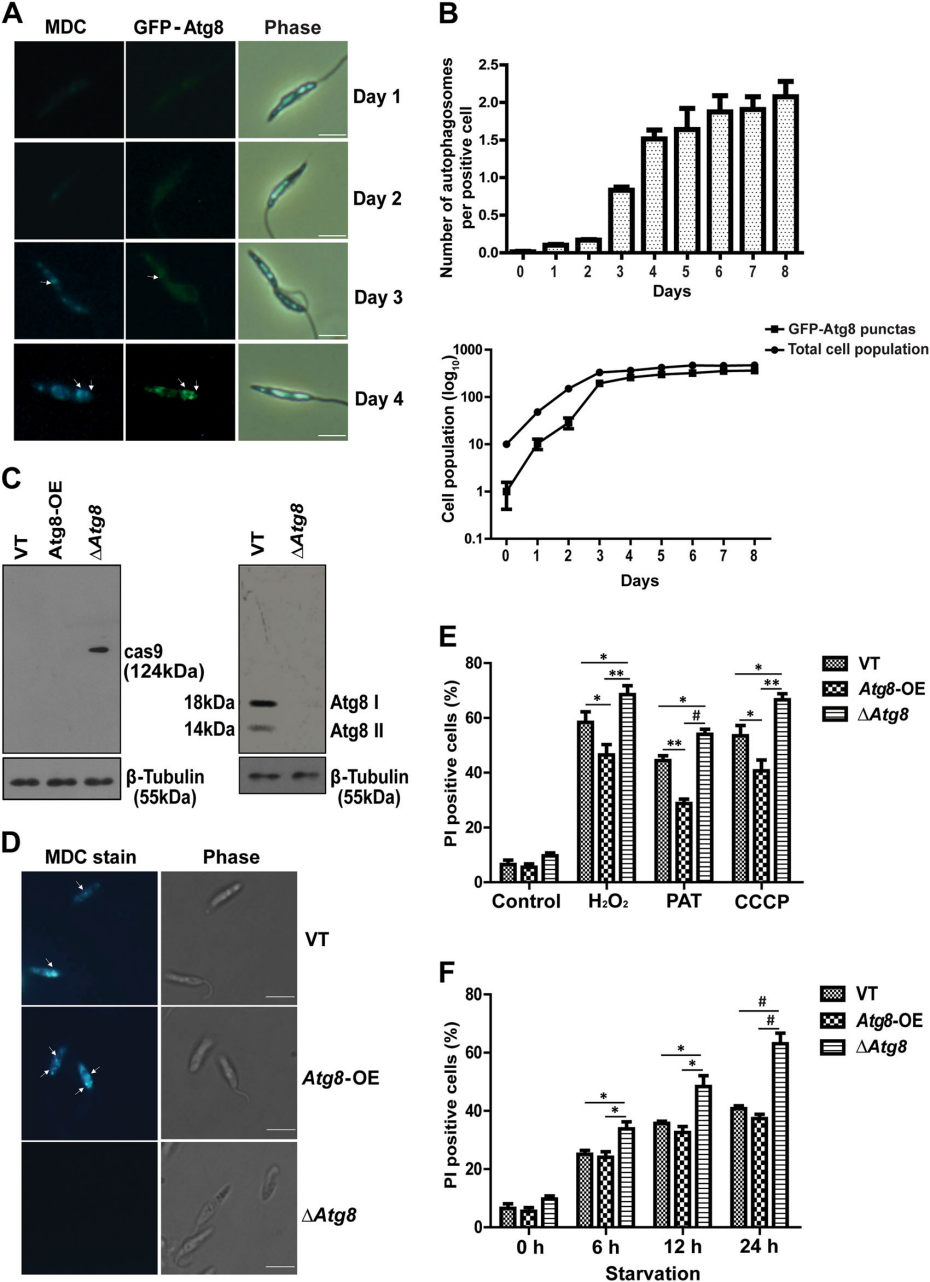
∆*Atg8* parasites are more vulnerable to drug-induced stress. **a** Parasites overexpressing GFP–Atg8 (*Atg8*-OE) protein were grown under normal culture conditions and stained with MDC. An increase in MDC-positive vacuoles with an increase in days of culture, coincided with the increase in the number of GFP–Atg8 puncta. Arrows indicate autophagosomes. Scale bars represent 5 µm. **b** The upper panel indicates the number of autophagosomes per promastigote during growth in vitro. Data represent mean ± SEM (*n* = 3). The lower panel indicates the increase in the proportion of parasites with GFP–Atg8 punctas on a daily basis during the in vitro culture of parasites. Data represent mean ± SEM (*n* = 3). **c** Western blot analysis was carried out to confirm Cas9 overexpression and Atg8 levels in ∆*Atg8* parasites. **d** Late log-phase VT; *Atg8*-OE parasites showed significant MDC staining compared with ∆*Atg8* parasites. **e** Parasites treated for 12 h with indicated concentrations of H_2_O_2_ (100 µM), PAT (250 µM), and CCCP (200 µM) resulted in increased cell death in ∆*Atg8* parasites as compared with VT and *Atg8*-OE parasites. Data represent mean ± SEM (*n* = 3), *P < 0.05, **P < 0.005. **f** Parasites were cultured in media deprived of FCS for the indicated time periods, and high percentage of cell death was observed in starved ∆*Atg8* parasites as compared with VT and *Atg8*-OE parasites. Data represent mean ± SEM (*n* = 3), *P < 0.05, ^#^*P* < 0.0005

Autophagy is governed by a set of proteins called Atg proteins, and the *Leishmania* spp. expresses several orthologs of mammalian Atgs^9^. An increase in *Atg5, Atg8*, and *Atg12* transcript levels as compared with controls was recorded during stress exposure (Figs. 1g, S1). Given the fact that *Atg8* is central to the maturation of autophagosomes^9,10,25^, we further probed the functional role of this gene product*. Atg8* is a homolog of *LC3* gene of higher vertebrates. Cytoplasmic LC3 protein gets cleaved into truncated LC3II enabling the formation of a stable association with membrane-bound phosphoethanolamine, thus regulating the crucial terminal stages of autophagosome formation^7,9,10^. *L. donovani* expresses two copies of *Atg8* genes on chromosome 19 (Ld_190840 and Ld_190850). The full-length *Atg8* gene Ld_190840 was targeted in this study. To study changes in Atg8 protein during the in vitro life cycle*, Atg8* gene fused to GFP was overexpressed in log-phase parasites (*Atg8-*OE) by using an episomal expression system (Fig. S1). In the overexpressing parasites (plated at a concentration of 10^6^ cells per ml), there was a progressive increase in GFP expression, initially visible as diffused staining in the cytoplasm, and as the life cycle progressed, GFP punctas were detected (Fig. 2a). Evaluation of the number of MDC-stained autophagosomes per cell showed a progressive increase with the cells entering the stationary phase of the life cycle (Fig. 2b). This corroborated with the pattern of increased occurrence of GFP–Atg8 punctas in the same stage of parasites’ life cycle (Fig. 2b).

Ideally, if Atg8-regulated autophagy was essential for the parasites to survive, deprivation of Atg8 protein would have a profound impact on the survival of the parasites. CRISPR–Cas9 technology was used to generate deletion mutants of *Atg8* (Δ*Atg8*) where part of the gene was disrupted (Fig. S2A–D) to stop Atg8 protein synthesis. Vector-transfected cells (VT) were used as controls. Extracts of Δ*Atg8* parasites showed no expression of the Atg8 protein as compared with VT parasites (Fig. 2c). MDC staining confirmed a higher level of autophagy in GFP–*Atg8*-OE and VT parasites, comparatively, much more than Δ*Atg8* parasites (Fig. 2d). A comparison of survival between VT, *Atg8*-OE and the Δ*Atg8* parasites during stress clearly demonstrated increased vulnerability of Δ*Atg8* parasites to H_2_O_2_, PAT, and CCCP-induced stress (Fig. 2e). Similarly, starvation-induced death was higher among Δ*Atg8* parasites as compared with VT or the *Atg8*-OE groups (Fig. 2f). Therefore, it was evident from the above studies that the presence of the Atg8 protein was required for survival under stress.

### Absence of Atg8 protein interferes with differentiation to axenic amastigotes

**Fig. 3:**
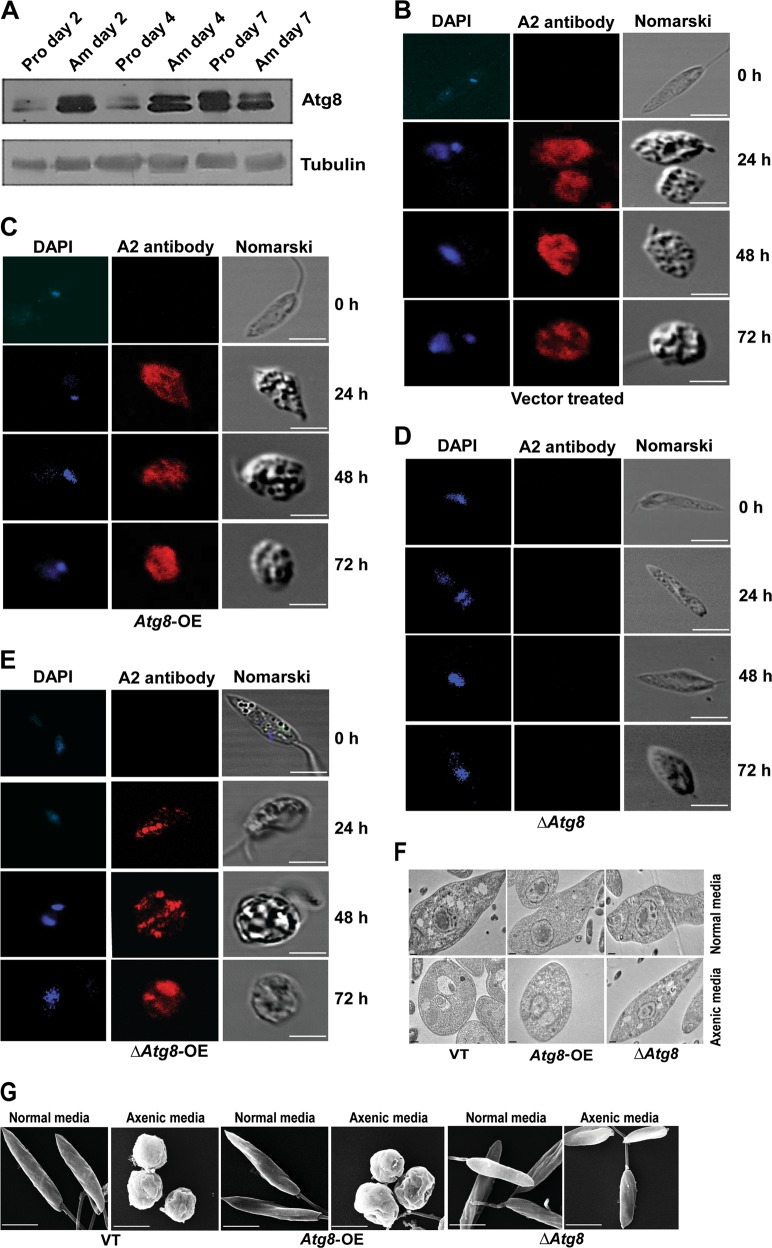
∆*Atg8* parasites were unable to differentiate. **a** Atg8 protein expression was determined on day-2, -4, and -7 amastigotes (isolated from macrophages) and in in vitro culture days of 2, 4, and 7 of promastigotes, where tubulin blots were used as loading controls. Conversion assay of promastigotes to amastigotes (grown in axenic media) by using **b** VT, **c** *Atg8*-OE, **d** ∆*Atg8*, and **e** ∆*Atg8*-OE parasites was assessed by A2 antibody staining (A2 being specific for amastigotes). Note that ∆*Atg8* parasites were negative to A2 staining and also retained an elongated shape as opposed to nearly spherical shape of amastigotes. For **b**–**e**, scale bars represent 5 µm. **f** Transmission electron micrographs showed elongated structures of promastigotes in normal media, whereas the conversion to amastigotes is seen with axenic media in the case of VT and *Atg8*-OE parasites. ∆*Atg8* parasites show an elongated promastigote-like structure even under axenic conditions. Scale bars represent 500 nm. **g** Scanning electron micrographs showing morphological features of VT, *Atg8*-OE, and ∆*Atg8* parasites. Scale bars represent 5 µm

While survival under stress is one aspect of parasite physiology, the ability to infect the host macrophages is an essential function. After the *Leishmania* parasite infects host macrophages, they change shape to a rounded disease causing amastigote form capable of surviving within host phagolysosomes^26^. This differentiation involves substantial cellular remodeling including a reduction in size; therefore, the involvement of processes like autophagy is expected to be essential during this period. Promastigotes of days 2 and 4 of culture expressed low levels of Atg8 as compared with late stationary phase (day 7), and the amastigotes recovered from infected host macrophage cell lines (THP1) (Fig. 3a), showing an association of Atg8 expression with differentiation of the parasite. Therefore, to investigate if Atg8 was essential for promastigote-to-amastigote differentiation, we used the in vitro system of culturing parasites for the generation of axenic amastigotes at 37 °C and pH of 5.5 (axenic media). While the VT (Fig. 3b, f, S3A) and *Atg8-*OE (Fig. 3c, f, S3A) parasites started to round up in axenic media at 24 h, being well rounded at 72 h of culture, the Δ*Atg8* parasites did not show any change in shape and remained elongated (Fig. 3d, f, S3A). The amastigotes of VT and *Atg8-*OE cells were positive for anti-A2 antibody staining (Fig. 3b, c), a marker for amastigote-stage parasites^27^. The Δ*Atg8* parasites were negative for anti-A2 antibody stain (Fig. 3d) showing repression of differentiation to amastigotes. Restoration of *Atg8* function by overexpressing *Atg8* in Δ*Atg8* cells (Δ*Atg8*-OE) with codon-optimized mutant (Fig. S3B) (such that the target sites for gRNAs are altered without loss of function), could revive the parasites’ ability to differentiate^27^ by changing into rounded forms that were positive for anti-A2 antibody staining (Fig. 3e). These data therefore provide evidence for suppression of differentiation to amastigotes in the absence of Atg8 protein pointing toward a crucial function being regulated by Atg8. Transmission electron photomicrographs of the three groups of parasites (VT, *Atg8*-OE, and Δ*Atg8*) clearly illustrate the inability of Δ*Atg8* parasites to differentiate into rounded amastigote forms when grown in axenic media in vitro (Fig. 3f). Further proof of this inability to differentiate is also shown in scanning electron micrographic images of VT, *Atg8-*OE, and Δ*Atg8* parasites grown in axenic media (Fig. 3g).

### Absence of Atg8 protein reduces the parasites’ capability to infect macrophages

**Fig. 4:**
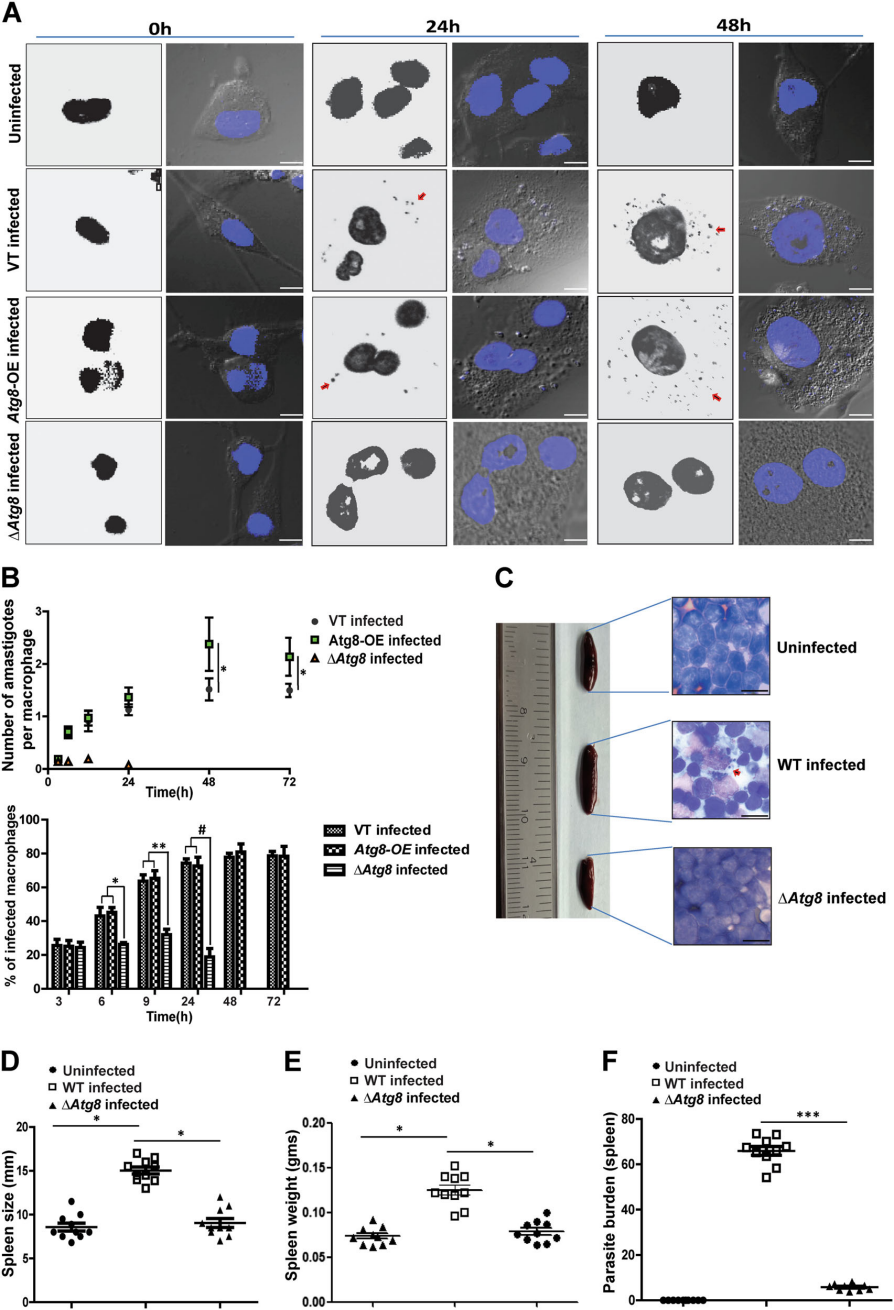
∆*Atg8* parasites were unable to establish infection. **a** THP1 monocyte-derived macrophages infected with *L. donovani* show the highest infection rates with *Atg8*-OE as compared with ∆*Atg8* and VT. Arrows indicate the amastigotes within the infected macrophages. Scale bars represent 10 μm. **b** The upper panel shows the number of amastigotes/macrophage. Data represent mean ± SEM (*n* = 3, from counts of 500 macrophages), *P < 0.05. The lower panel shows changes in the percentage of infected macrophages with respect to the duration of infection. Data represent mean ± SEM (*n* = 3), *P < 0.05. Note that infection by ∆*Atg8* parasites is low. **c** Data from spleens (*n* = 10) of uninfected mice and those infected with ∆*Atg8* parasites showed a significant difference in spleen size between WT-infected and ∆*Atg8-* infected animals. Photomicrographs of splenic imprints show parasite load in mice infected with WT and ∆*Atg8* parasites. **d**, **e** Size and weight of mice spleen isolated from *Leishmania-*infected samples. Increase in weight and dimensions of spleens are evident in mice infected with WT parasites compared with those infected with ∆*Atg8* parasites in which infection was negligible, *n* = 10. **f** Parasite burden in terms of Donovan units was calculated from splenic smears. LDU, Leishman–Donovan units, *n* = 10

In the backdrop of the above data and the increased expression of Atg8 protein in amastigotes recovered from macrophages, it was hypothesized that growth and survival within the mammalian host cells could be dependent on Atg8. To explore such a possibility, human macrophages differentiated from a THP1 monocyte cell line were infected with VT, *Atg8-*OE, and Δ*Atg8* parasites. Both the VT and *Atg8-*OE parasites at 24 and 48 h of infection showed significant propagation of amastigotes, whereas the Δ*Atg8* parasite-infected macrophages were devoid of amastigotes (Fig. 4a, b). This observation conforms to our findings of the inability of Δ*Atg8* to convert to amastigotes. Further confirmation of the Δ*Atg8* parasites being unable to sustain as amastigotes comes from the in vivo studies where animals infected with Δ*Atg8* parasites showed no enlargement of spleen in a mouse model of leishmaniases as compared with larger spleen size and spleen weight of WT parasites (Fig. 4c–e). Also, WT parasites showed much higher parasite load in splenic smears as compared with smears from spleens of animals infected with Δ*Atg8* parasites (Fig. 4f). In summary, both in vitro and in vivo studies bear evidence for the necessity of Atg8 for parasite survival within macrophages.

### Mitochondrial translocation of Atg8 occurs during differentiation to amastigotes

**Fig. 5:**
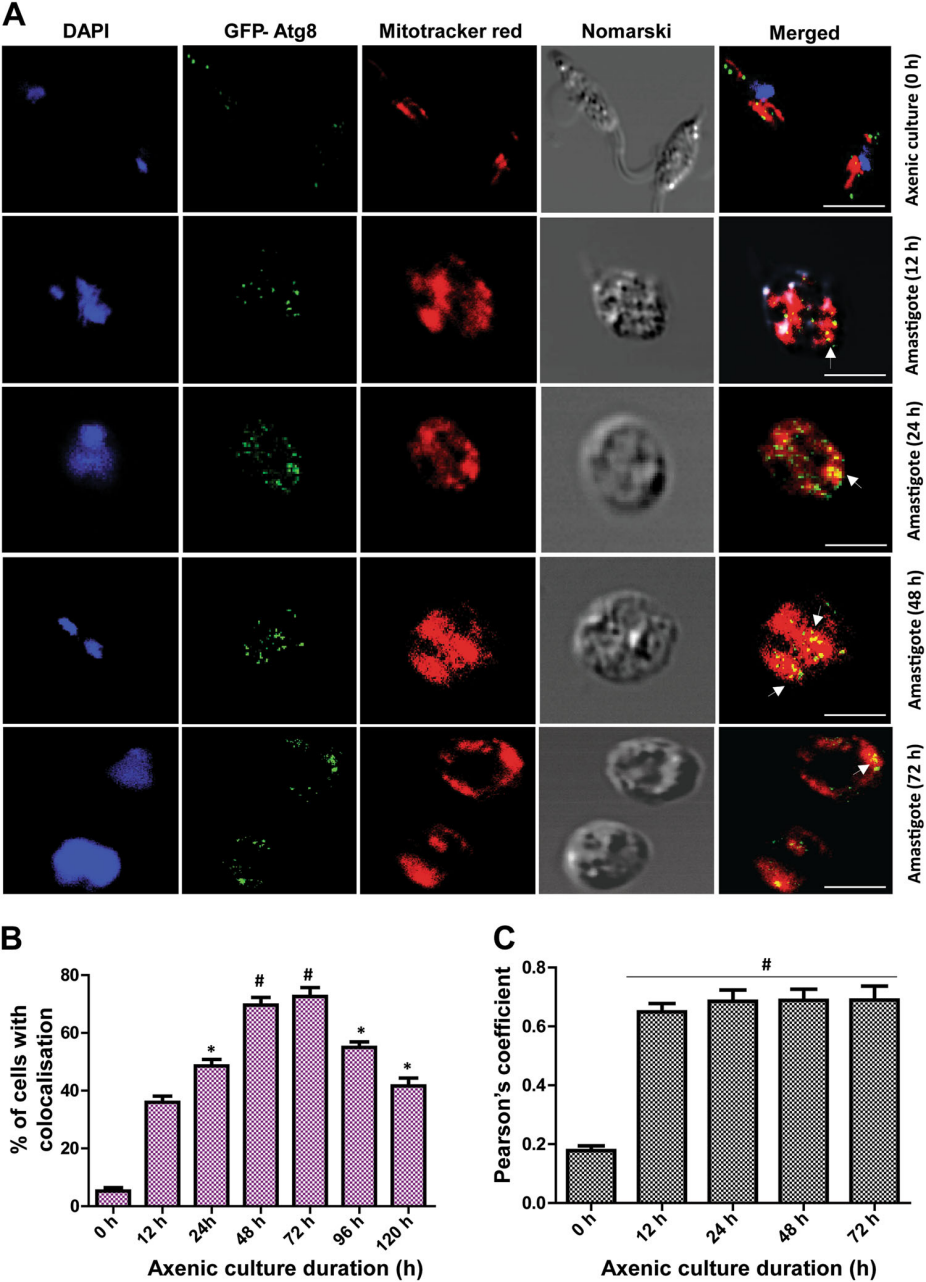
Atg8 protein migrates to the vicinity of mitochondria during differentiation. **a** *Leishmania* promastigotes expressing GFP–Atg8 were cultured in axenic media for conversion to amastigotes and stained with Mitotracker® Red. Subcellular localization of GFP–Atg8 punctas was followed, and the increasing presence of the GFP–Atg8 punctas in the vicinity of the mitochondria was visible. Data shown are representative of three experiments. Scale bars represent 5 µm. **b** Localization index with Pearson’s coefficient exceeding 0.6 was considered to be positive, and the percentage of cells with positive localization coefficient during differentiation is shown. Data represent mean ± SEM (*n* = 3), *P < 0.01, ^#^*P* < 0.001. **c** A constructed bar graph of Pearson's coefficient. Data represent mean ± SEM (*n* = 5), ^#^*P* < 0.01

Conversion to amastigotes involves significant cellular remodeling that prompted us to track the changes in Atg8 localization during conversion to axenic amastigotes. For this, promastigotes grown in axenic culture media were allowed to differentiate into axenic amastigotes. *Atg8-*OE parasites expressing GFP showed a punctate localization of GFP on the mitochondria stained with Mitotracker® Red at 12, 24, 48, and 72 h of axenic amastigote differentiation, suggesting the translocation of Atg8-positive autophagosomes near the mitochondria, indicating autophagic activity in the vicinity of the organelle (Fig. 5a). Similar activity occurs in mammalian cells where damaged mitochondria are removed by an autophagic process termed “mitophagy”^28^. Although the process of mitophagy has not been described in these parasites, the translocation of Atg5 protein to the mitochondria was described earlier in *L. major*^11^. In our studies, maximum accumulation was observed during differentiation with an upward trend till 72 h, reducing thereafter once the entire population was converted to amastigotes (Fig. 5b). The coefficient of colocalization was taken into consideration for the analysis (Fig. 5c) where the movement of Atg8 toward the mitochondria was high 12 h onward. This is similar to one of the prominent evidence of changes in subcellular localization of Atg8/LC3II in mammals observed during mitophagy where the Atg8/LC3II protein localizes onto the damaged mitochondria helping to form autophagosomes for mitophagy^20,29^. These studies therefore show increased autophagic activities near the mitochondria.

### Atg8 accumulation near the mitochondria is linked to oxidative stress

**Fig. 6:**
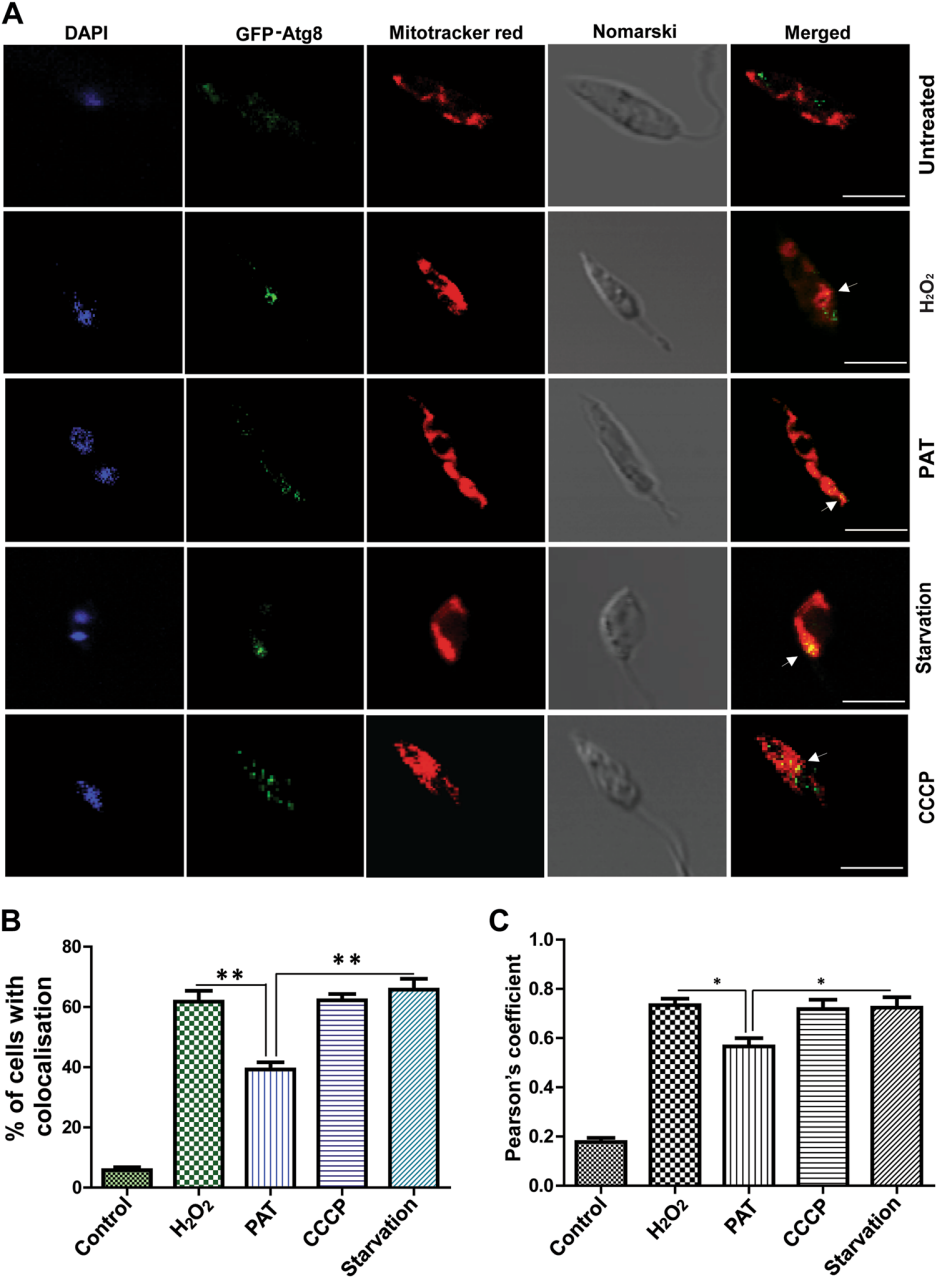
Atg8 migrates near the mitochondria in response to drug stress. **a** The position of GFP–Atg8 punctas with respect to Mitotracker® Red was assessed in parasites treated with H_2_O_2_, PAT, and CCCP for 6 h. Starved cells were used as positive controls. Arrows indicate the possible site of contact of autophagosomes and area surrounding the mitochondria. **b** Representation of the percent of cells with mitochondrial co-localization of Atg8 protein after drug treatment and starvation. Data represent mean ± SEM (*n* = 3), *P < 0.05, **P < 0.005. **c** A constructed bar graph of Pearson's coefficient. Data represent mean ± SEM (*n* = 5), *P < 0.05

GFP-tagged *Atg8-*OE cells treated with oxidative stress inducers like H_2_O_2_, CCCP, and PAT showed buildup of GFP puncta near the mitochondria (Fig. 6a). Earlier studies by using *L. major* showed translocation of Atg5-associated autophagosomes to parasite mitochondria on starvation^11^. In our studies, starvation was used as a positive control. Further analysis of z-stacked images of drug-stressed parasites by IMARIS software also resonated the observation of migration of Atg8 onto the vicinity of mitochondria (Fig. S4A). The percentage of cells with accumulation of Atg8 near the mitochondria was higher with H_2_O_2_, CCCP, and starvation-induced stress as compared with PAT-induced stress (Fig. 6b). Statistical analysis of Pearson’s coefficient values also reflected less mitochondrial accumulation of Atg8 in PAT-treated cells (Fig. 6c). Interestingly, increases in superoxide/reactive oxygen species levels were noted with H_2_O_2_, CCCP treatment, and starvation that were higher than PAT- treated cells (Fig. S4B). In summary, Atg8-positive autophagosomes were found to translocate to the region near the mitochondria in response to drug-induced stress and starvation.

### Mitotoxic but not genotoxic stress triggers autophagosome translocation to mitochondria

As evident from our previous data, mitochondrial accumulation of Atg8 was observed in a larger population of cells under the influence of stress generators, with the exception of PAT-treated parasites. Our results also revealed PAT to be a weaker superoxide/reactive oxygen species inducer compared with the other drugs used. This led us to the question whether the accumulation of autophagosomes was responsive to mitotoxic stress only, where mitochondrial damage is intensified by generation of superoxides/reactive species.

**Fig. 7:**
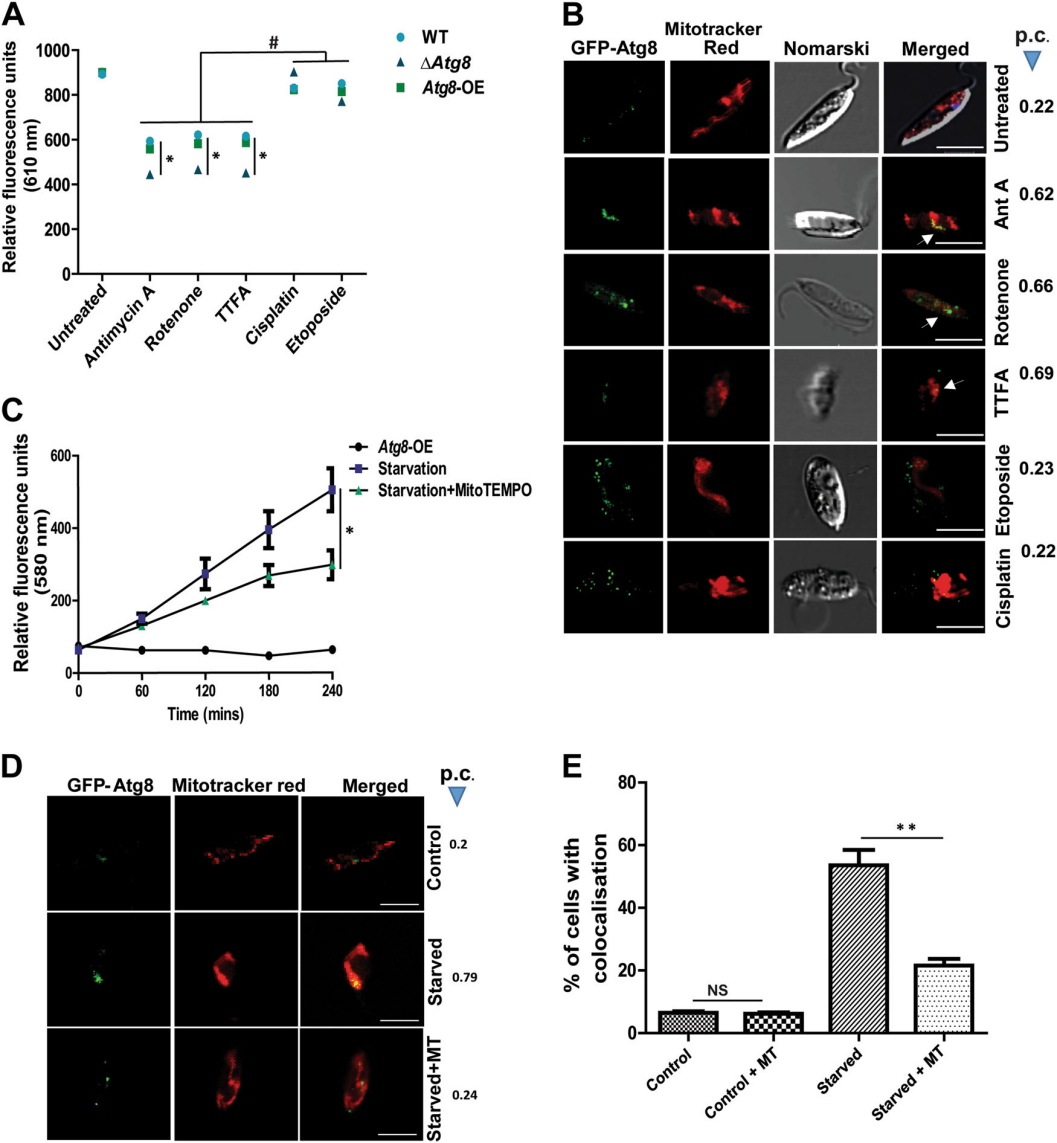
*Atg8* migrates to the mitochondria in response to mitochondrial stress and not genetic stress. **a** Parasites were treated with mitochondrial depolarizing agents (antimycin A, rotenone, and TTFA) and genetic stressors (cisplatin and etoposide), and the change in mitochondrial potential was assessed by staining with potentiometric dye Mitotracker® Red after respective treatments for 2 h. Note that parasites treated with mitotoxic agents show significant decline in mitochondrial potential. Error bars represent  ± SEM (*n* = 3), *P < 0.05 represents the difference in mitochondrial potential between WT and ∆*Atg8* parasites treated with mitotoxic agents; ^#^*P* < 0.0005 represents the difference in potential between the groups of WT parasites treated with mitotoxic and genotoxic drugs. **b** Mitochondrial translocation of Atg8 in *Atg8*-OE cells was assessed in the treated groups by tracking the GFP–Atg8 puncta and staining the mitochondria with Mitotracker® Red. Localization index was statistically analyzed by considering the values of Pearson’s coefficient. Note low co localization in the case of genotoxic response. **c** Atg8 parasites starved in the presence of MT and stained with MitoSOX™ Red, show superoxide**/**ROS generation where MT was able to reduce superoxide/ROS production. Data represent mean ± SEM (*n* = 3). **d** Position of Atg8-associated punctas with respect to Mitotracker® Red stained mitochondria in starved parasites were assessed and compared with those treated with MT. Reduction of migration of GFP–Atg8 was observed under the influence of MT. **e** Comparative analysis highlights the inhibition of GFP–Atg8 translocation to mitochondrial vicinity under the influence of MT. Data represent mean ± SEM (*n* = 3), *P < 0.01, NS, nonsignificant

To generate mitochondrial stress, we used three mitochondrial respiratory chain inhibitors, Antimycin A (Complex-III inhibitor), Rotenone (Complex-I inhibitor), and TTFA (Complex-II inhibitor) to induce mitochondrial damage and investigated the accumulation of autophagosomes near mitochondria. Two genotoxins, cisplatin and etoposide, were also used for comparative analysis. Analysis of mitochondrial potential with JC-1 dye in wild-type cells indicated that genotoxic drugs do not affect the mitochondrial potential during the initial hours of treatment, and a drop in mitochondrial potential is evident only in the case of prolonged treatment (Fig. S6A). In *Atg8-*OE cells, Mitotracker® Red (a potentiometric red fluorescent dye that accumulates in the mitochondria of viable cells) was used to measure mitochondrial potential. A fall in mitochondrial potential was recorded after treatment with the mitochondrial respiratory chain inhibitors but not with cisplatin and etoposide, the genotoxins (Fig. 7a). Mitochondrial mass is typically defined as an increase in bulk of the mitochondria^30^. MitoTracker™ Green FM, which does not fluoresce in aqueous solutions, but becomes fluorescent at accumulation in the mitochondrial lipid environment, was used to measure mitochondrial mass. The mitochondrial mass of the parasites treated with cisplatin and etoposide was found to be unaffected as compared with the increased masses of those treated with antimycin A, rotenone, and TTFA (Fig. S6B). Parasites with mutated Atg8 showed increased mitochondrial mass and decreased mitochondrial potential compared with the others under the influence of mitotoxic agents, indicating their vulnerable mitochondrial health (Figs. 7a, S6B). Figure 7b shows Atg8 translocation to the mitochondria in *Atg8-*OE parasites exposed to respiratory chain inhibitors as opposed to the same parasites subjected to genotoxic stress where translocation was negligible. This suggested that changes in subcellular localization of Atg8- positive autophagosomes toward the mitochondrial vicinity, largely occur in response to mitochondrial damage.

The above data led to the question as to what could act as a signal in response to mitochondrial damage to draw Atg8-positive autophagosomes to the mitochondria. Prior knowledge shows superoxide increases with mitochondrial damage^22,23^; therefore, it was pertinent to generate superoxide to see if the translocation occurred. We used MitoSOX™ Red^31^, known to measure superoxide although a few studies show that it can detect other species of reactive oxygen^32^, so henceforth we have referred to these measurements as superoxide/ROS. *Atg8-*OE cells were subjected to starvation in incomplete media, and superoxide/ROS was measured by using MitoSOX™ Red^31^ where there was an elevation of superoxide/ROS levels that could be scavenged by Mito-TEMPO (MT)^33,34^ (Fig. 7c). MT is a mitochondria-targeted superoxide dismutase mimetic that holds superoxide and alkyl radical-scavenging activity^35^. Scavenging of superoxide/ROS by MT resulted in the recovery of mitochondrial potential (as observed in starved WT parasites, Fig. S7A) and mitochondrial mass (Fig. S7B). An increase in superoxide/ROS levels in starved cells was accompanied by increased co-localization of Atg8 puncta with Mitotracker® Red, but exposure to MT reduced the co-localization (Fig. 7d, e), suggesting a reduction in Atg8 accumulation upon quenching of superoxide/ROS. Therefore, data suggest the possibility that the accumulation of the Atg8 on the mitochondria largely depends upon superoxide/ROS generation by anomalous mitochondrial activity.

## Discussion

Autophagy is a crucial process in protozoan parasite survival as indicated in earlier studies with *Leishmania, Plasmodium*, and *Trypanosoma* spp.^9,36,37^. Prior studies with the trypanosomatid parasite *L. major*, the causative agent for cutaneous leishmaniasis, established the importance of autophagy during its life cycle^6^. Data presented here with another *Leishmania* species, *L. donovani* that causes the visceral form of the disease, corroborate the findings on the essential nature of autophagy in *L. major*. Like higher eukaryotes, the control of molecular machinery of autophagic activities in *Leishmania* spp. comprises multiple Atg proteins^10,25^. After genome sequencing of *Leishmania* spp., several mammalian orthologs of *Atg5, Atg8, Atg12*, and *Atg16* genes were annotated^9^. Mottram and coworkers demonstrated the crucial role of the Atg5–Atg12 conjugation pathway in *L. major* parasites where mutants deleted for the Atg5 gene were unable to form autophagosomes^11^. Atg8 is a key autophagy protein participating in autophagosome formation through a unique ubiquitin like conjugation to phosphatidylethanolamine on the autophagic membrane. Eventually, Atg4 helps in delipidation of Atg8 attached to the outer autophagosomal membrane facilitating recycling^38^. The role of Atg8 in lower eukaryotes has been previously shown in *Saccaromyces cerevisae*^39^. A study in the related protozoan parasite, *Plasmodium falciparum*, has suggested that *Pf*Atg8 may be involved in endoplasmic reticulum (ER)-related organelle biogenesis, including apicoplast membrane biogenesis^40^. The involvement of Atg8 in Atg5–Atg12 conjugation pathway to facilitate phospholipid homeostasis through autophagy in *L. major*^11^ has been recently studied. However, its role in response to stress and infectivity remains unexplored. The studies described here provide evidence for the involvement of Atg8 protein in *L. donovani* differentiation to amastigotes, sustenance in host cells, and a possible contribution in the maintenance of mitochondrial homeostasis.

Upregulation of *Atg5*, *Atg8*, and *Atg12* genes linked to the autophagy pathway accompanied by an increase in MDC-positive vacuoles after stress exposure observed in this study, clearly indicated a positive autophagy response. The necessity of functional Atg8 protein for resistance to stress was shown in experiments where Δ*Atg8* mutants with deleted *Atg8* gene demonstrated greater susceptibility to stress in comparison with wild-type parasites when exposed to mitochondrial respiratory chain uncouplers, oxidative stress, or the anti-leishmanial drug potassium antimonyl tartrate. On the contrary, overexpression of the *Atg8* gene in WT cells (*Atg8-*OE) or in *Atg8*-silenced cells (Δ*Atg8-*OE), made the parasites more resistant to stress- induced death confirming the involvement of Atg8 in successful stress resistance. In contrast to reports on *Atg5* mutants where the parasites showed morphological abnormalities^11^, the Δ*Atg8* did not show morphological abnormalities but was sluggish and slower in progressing through the life cycle suggesting an alteration in metabolic activities (Fig. S6). The growth rates of the mutant parasites were compromised as they progressed through late log phase, probably showing effects of the absence of the Atg8 protein on parasite growth. Other than gene deletion, blocking of autophagy in wild-type parasites by using an autophagy inhibitor 3-MA resulting in low viability during differentiation in vitro and under stress was also evidence for the important role that autophagy plays.

The differentiation of promastigotes to amastigote forms in mammalian macrophages and their survival within the host cells are the primary requirements for the onset of the disease^13^. The failure of Δ*Atg8* parasites to differentiate into axenic amastigotes in vitro as opposed to VT and *Atg8-*OE cells confirmed involvement of Atg8 protein in differentiation to disease-causing amastigotes. Since conversion of promastigotes to amastigotes involves considerable cellular remodeling^13^, it is possible that compromising autophagy becomes a hindrance for completion of proper reshaping. This is important because the ability of the parasites to convert to amastigotes is most essential to generate successful infection. The poor survival of the Δ*Atg8* parasites within macrophages after infection corroborates with the observation of their inability to differentiate in vitro. Understandably, changes in shape and cell size accompanied by alterations in organelles as well, are major events during differentiation^12,13,26^, and therefore it is possible that lack of Atg8 prevented those events, hindering conversion to amastigotes. The in vitro infection studies were supported by in vivo observations in a mouse model of leishmaniases where splenic enlargement with sufficient parasite load in splenic aspirates was seen in control mice infected with WT parasites but were absent in those infected with Δ*Atg8* parasites. Interpretation of the above data supports the notion that Atg8 protein plays an important role in contributing to the infective abilities of the parasite.

The single mitochondrion of the *Leishmania* parasite is large and is distributed throughout the body of the parasite^41^. In contrast, mammalian cells possess many mitochondria and their turnover is taken care of by the process of mitophagy, which is a form of selective autophagy^42^. Mitophagy is not reported in *Leishmania* spp., however, accumulation of autophagic vacuoles around *L. major* mitochondria was shown under starvation^11^. In this study, starvation-induced GFP-tagged Atg8 relocation to the mitochondrial vicinity in *Atg8*-OE parasites showed similarity to the *L. major* Atg5 studies^11^. Also, when challenged with both exogenous and endogenous stress-inducing drugs, GFP–Atg8 punctas tended to localize near the mitochondria. Future studies can shed light on the consequences of this close association as to the possibilities of mitophagy-like activities. Interestingly, a comparative analysis of mitochondrial accumulation of Atg8 in parasites treated with mitotoxic stressors with those challenged with genetic stressors suggested that the event could be initiated in response to mitochondrial changes like a drop in potential or an increase in mass but not by DNA damage. Since scavenging of mitochondrial superoxide attenuated accumulation of GFP–*Atg8* punctas on mitochondria along with recovery of mitochondrial potential and mass to near-normal levels, the importance of superoxide/ROS in the regulation of mitophagy-like activities is a strong possibility. The scavenging of superoxide/ROS generated in the mitochondria, leading to lesser accumulation of the Atg8 puncta during starvation, also suggests a stress sensor-like function of the superoxide/ROS. As evident from this study, loss of mitochondrial potential leads to accumulation of Atg8-associated autophagosomes in close vicinity of mitochondria. Vulnerability of Δ*Atg8* parasites in response to oxidative stress indicates that the above-said accumulation event probably assists survival of the parasites. Very little is understood about the mitochondrial biochemistry of this parasite, and it can be speculated at this stage that some form of autophagy in selective areas of the mitochondria may occur.

The emergence of resistant strains of the parasites illuminates the necessity to discover new drug targets. The process of autophagy, which is important for survival, infection, and growth, is a convenient target of interest. A deeper understanding of autophagy-related events in protozoan parasites and targeting them would open up avenues for intervention of parasite growth. Of particular relevance is a recent study where a class of compounds have been identified to inhibit *Plasmodium* Atg8–Atg3 interaction and presumably *Pf*Atg8 lipidation, eventually attenuating the growth of blood- and liver-stage parasites^43^. Similar studies can be done with *Leishmania* as model parasites. However, the development of future methodologies would necessitate exercising a balance of careful data evaluation and application. In a nutshell, this study provides evidences for involvement of Atg8 in the vital processes linked to *Leishmania* survival.

## Materials and methods

### Ethics statement

All animal experiments were duly approved by the Institutional Animal Ethics Committee of the National Institute of Immunology, New Delhi (IAEC/AQ/2019/173, serial no. IAEC#489/18).

### Animals

For in vivo experiments, inbred mice of C57BL/6 background of either sex at 6–10 weeks of age were used. Animals of the same age were randomly selected from the large group of C57BL/6 mice repository of the institute. Sample size was calculated manually by using the formula of sample size/[1−(%attrition/100)], where attrition was expected to be 10% of the sample size.

### Cells

A strain of *L. donovani*, BHU1260, was derived from the splenic aspirate of a VL patient at the Kala Azar Medical Centre of the Institute of Medical Sciences, Banaras Hindu University, Varanasi, India^44^. The culture was maintained in Medium 199 (M199) supplemented with 10% fetal calf serum (FCS) at 25 °C. Axenic amastigotes were generated by culturing the promastigotes from log-phase culture in M199 at 37 °C and pH 5.5^45,46^. Starvation was induced in the parasites by removing them from FCS- fortified media, washing, and culturing in media without FCS. The human monocytic cell line THP1 (ATCC TIB-67) was maintained at 37 °C and 5% CO_2_ in Roswell Park Memorial Institute Medium (RPMI; Sigma-Aldrich, St. Louis, MO) containing sodium bicarbonate (350 mg·ml^−1^), glucose (4.5 g·ml^−1^), and 10% FCS.

### Plasmids

Overexpression of full-length Atg8 protein (Ld_190840) in the parasites was carried out by using pXG-GFP+2 vector (a kind gift from Dr. Stephen Beverley) as described earlier^47^. For making deletion mutants by CRISPR–Cas9, plasmids from Greg Matlashewski’s laboratory^48^ as available in Addgene were used. Cas9 was expressed by using pLPhygCAS9 construct, and the guide RNAs against *Atg8* were cloned in pSPneo HHgRNAH vectors^48^. In *Atg8*-deleted mutants (∆*Atg8*), *Atg8* was complemented by overexpressing mutated *Atg8* by using pXG-GFP+2 vectors to generate ∆*Atg8-*OE parasites.

### Cell viability

To measure cell viability, Propidium Iodide (PI, Qiagen, St. Louis, MO) assay was carried out. Briefly, after treatments, parasites were washed and incubated with 50 µg·ml^−1^ of PI for 10 min. The cell population was analyzed for PI staining by using flow cytometry with a BD FACS Calibur (BD Biosciences, San Jose, CA) as described previously^49^.

### Production of Atg8 antibody

*L. donovani Atg8* gene was inserted into pET-28 a (+) expression vector with histidine residues (His tag) at the N-terminal end. The recombinant vector was then transformed in *Escherichia coli* BL-21 strain. The total expressed protein of the bacteria was recovered by sonication. Thereafter, the cytosolic Atg8 protein was purified by affinity chromatography against His tag at the N-terminal end by using nickel-NTA (Ni^2+^-NTA) beads (Qiagen, Hilden, Germany). *L. donovani*-specific anti-Atg8 antibody was prepared by subcutaneous dosages of 0.5 purified Atg8 protein emulsified with Complete Freund’s Adjuvant (CFA, 100 µg·ml^−1^) in New Zealand White rabbits. The dosage was repeated with incomplete Freund’s adjuvant (IFA, 100 µg·ml^−1^) after an interval of 14 days followed by the second booster dose on day 28^50^. On day 35, the first bleed was collected (~25 ml) followed by the third booster on day 42. The second and the third bleeds were collected on days 56 and 58. The third bleed was used for the studies.

### Drugs and chemicals

To induce stress conditions in the parasites, various stress-inducing drugs were used like hydrogen peroxide (H_2_O_2_, Merck, India, 100 µM), potassium antimonyl tartrate (PAT, 250 µM), and carbonyl cyanide m-chlorophenyl hydrazine (CCCP, 200 µM). Mitochondria-specific stress was generated by the use of specific respiratory chain uncoupling agents like antimycin A (mitochondrial complex-III inhibitor, 100 µM), rotenone (mitochondrial complex-I inhibitor, 100 µM), and thenoyltrifluoroacetone (TTFA, mitochondrial complex-II inhibitor, 200 µM) as described previously^51^. To generate genetic stress, etoposide, a topoisomerase II blocker (100 µM) and cisplatin, the anticancer drug (200 µM) were used. All drugs and chemicals were purchased from Sigma-Aldrich, St. Louis, MO, unless otherwise mentioned.

### Measurement of mitochondrial potential, superoxide content, and mitochondrial mass

To measure mitochondrial potential, potentiometric probe JC-1 (5,5′6,6′-tetrachloro-4421,1′3,3′-tetraethylbenzimidazolyl carbocyanine iodide) (Thermo Fisher Scientific; T3168) was used as described earlier^52^. Briefly, log-phase parasites at a concentration of 5 × 10^6^ cells ml^−1^ were treated with drugs for the specified period of time and then incubated with 5 µg·ml^−1^ of JC-1 for 30 min followed by washing with PBS, and fluorescence was measured by flow cytometry. Green and red fluorescence were measured by using 488-nm excitation with 530- and 585-nm band-pass emission filters, respectively. GFP-expressing cells could not be assessed for green fluorescence due to interference. Total intracellular ROS was measured by 2′,7′-dichlorodihydrofluorescein diacetate (H_2_-DCFDA) (Thermo Fisher Scientific; D399) as described earlier^44,49^ (data not shown). Briefly, 5 × 10^6^ cells were suspended in complete media and incubated with 2 μg·ml^−1^ CM-H_2_DCFDA for 15 min, and the desired treatments were carried out followed by fluorescence assay by using BMG black 96-well plates measured by using a microplate reader (CLARIOstar, BMG Labtech) at an excitation/emission at 530–10/590–10 nm. Mitochondria-generated superoxide was measured with MitoSOX™ Red (Thermo Fisher Scientific; M36008), a probe for detection of superoxides in the mitochondria as described previously^44^. About 5 × 10^6^ parasites ml^−1^ were preincubated with 0.5 M MitoSOX™ Red in 1× PBS for 30 min at room temperature. After relevant drug treatment, the kinetics of fluorescence change was measured with excitation and emission wavelengths set at 488 and 580 nm, respectively. Mitochondrial mass was estimated by MitoTracker® Green FM (Thermo Fisher Scientific; M7514), a dye that accumulates in mitochondria by reacting with the free thiol groups of mitochondrial proteins, following the manufacturer’s protocol^53,54^. Briefly, 5 × 10^6^ parasites ml^−1^ were washed with 1× PBS after the respective drug treatment for the indicated time duration and then incubated with 200 nM of Mito Tracker® Green FM for 30 min at room temperature. After washing, fluorometric measurement was done at excitation/emission at 488 /520 nm, respectively, by using BMG LABTECH CLARIOstar fluorometer (BMG LABTECH, Ortenberg, Germany).

### Cytochemistry

To determine the localization of Atg8 in the Atg8-overexpressing (Atg8-OE) log-phase parasites, GFP staining was assessed through excitation/emission filters set at 488/510 nm by using a Leica SP5 confocal microscope. For staining of the mitochondria, Mitotracker® Red (Thermo Fisher Scientific; M7512), a potentiometric dye was used. After staining, the cells were plated on poly-L-lysine-coated coverslips and their nuclei were stained with DAPI (Sigma-Aldrich, D9542). The coverslips were mounted on slides by using mounting media Vectashield (Vector laboratories, H-1000). Staining was visualized by using a Leica TCS SP5II microscope (Wetlzar, Germany) by using parameters as described previously^49,54^. Briefly, Argon ion laser was used to illuminate the excitation filters of 405, 488, and 561 nm for the purpose. Images were captured in point-averaging mode at zoom value 5.0, by using Plan Apo oil objective. Approximately, equal values of system-optimized gain and offset values were used in all acquired images. Scattered dot plots with a defined Pearson’s correlation coefficient for colocalization were generated by using the software of Leica TCS LAS AF version: 2.7.3.9723 (Leitz, Germany). Events with a value of Pearson’s coefficients (p.c.) above 0.6 calculated after removal of basal noise were considered significant as positive instances of colocalization. P.C. values of random individual 20 cells from each experimental set were calculated. Data from three independent experiments were then used to determine the percentage of cells with positive colocalization. For statistical representation of p.c. values, the respective values of 20 random individual parasites from each experiment were considered, and their mean value was calculated. Mean values from five experimental replicates were then plotted in a graphical form of representation. The z-stacked images were further reinterpreted for colocalization by using the “IMARIS Microscopy Tracking Software” (version 7.6.0) (Oxford Instruments, Switzerland).

For immunostaining of amastigotes by anti-A2 antibody, parasites were differentiated under axenic conditions. After 24, 48, and 72 h of culture, they were collected by centrifugation (1300 × *g*, 10 min) by using Centrifuge 5810R (Eppendorf, Hamburg, Germany), washed with 1× PBS, and then fixed with 4% paraformaldehyde for 10 min. The fixed parasites were permeabilized with 0.25% Triton X prepared in PBS for 4 min. The parasites were washed with PBS and incubated with 1% BSA blocking solution for 1 h, followed by incubation with primary anti-A2 antibody (ab150344, Abcam, Cambridge, UK) at 1:250 dilution overnight at 4 °C. Cells were then incubated with Alexa Fluor 594 anti-mouse secondary antibody (A11032, Invitrogen, Rockford, IL) at 1:500 dilution for 1 h at room temperature. The cells were mounted with Vectashield (Vector laboratories, H-1000) onto slides for microscopy.

### Western blots

Western blotting was used to estimate changes in the levels of Atg8 protein during differentiation. Briefly, the parasites grown under normal and axenic conditions were collected at different time points, and whole-cell lysates were prepared by using 1 × Laemmli buffer containing 2% SDS, 10% glycerol, 0.1% 2-mercaptoethanol, and 100 mM Tris-Cl (pH 6.8). Protein estimation was done by using an upgraded version of Bradford Assay using the CBX assay kit (G. Biosciences, St. Louis, MO). Equal amount of protein samples were loaded and resolved on 15% SDS-PAGE gel and subsequently transferred onto nitrocellulose membrane via a wet-transfer apparatus (BIO-RAD, Hercules, CA) operating at 100 V for 1 h. Transblotted proteins were blocked by using 5% bovine albumin serum (BSA), and immunoblotting was done by using relevant primary (anti-*Ld*Atg8 antibody, 1:1000) and secondary (anti-rabbit IgG, 1:5000) antibodies. Blots were developed with enhanced chemiluminescence (ECL) by using Femto Lucent kit and autoradiography using X-ray film. Quantitation of the relative density of signals on immunoblots was done by using Lab Works Image acquisition and analysis software version 4.0.0.8 (Lab Works, Analytik Jena, Upland, CA) as described earlier^49^. The β-tubulin antibody (PA1-16947, Invitrogen, Rockford, IL) (1:1000 dilution) was used to identify the protein on western blots to normalize the level of expression profile of other proteins of relevance after densitometry of the blots. Anti-Cas9 antibody was obtained from Santa Cruz Biotechnology, Inc. (sc-517386, Dallas, TX).

### Autophagy measurement

The level of autophagy in the parasites was measured by staining the autophagosomes with Monodansylcadavarine (MDC), a fluorescent marker of autophagic vacuoles. In brief, the parasites were collected post drug treatment and washed with 1× PBS, after which they were stained with 5 mM of MDC for 10 min. After removal of stain and wash, they were loaded onto poly-L-lysine-coated coverslips and mounted on slides. A fluorescence microscope (Nikon Eclipse E600) was used to identify MDC staining by using excitation filter of 405 nm. Fluorometric analysis of MDC staining was done with excitation/emission at 350 /525 nm, respectively, by using BMG LABTECH CLARIOstar fluorometer (BMG LABTECH, Ortenberg, Germany). By using the data obtained from three replicates of 100 GFP–Atg8-overexpressing parasites, the proportion of autophagosomes bearing cells, as well as the number of these structures per cell was assessed.

### Quantitative analysis of gene expression by reverse transcriptase PCR

Total RNA was isolated from cells by using a SV total RNA isolation kit following the manufacturer’s protocol (Promega, Z3105). The respective cDNAs were synthesized by using the superscript III first strand synthesis system (Thermo Fisher Scientific). Gene expression analysis was done with RT-qPCR by using SYBR Green PCR master mix (4367659, Thermo Fisher Scientific) using oligo (dT) primers. Primers used for gene expression analysis are listed in Fig. S1.

### Generation of deletion mutants by CRISPR–Cas9 technique

*Atg8* deletion mutants (∆*Atg8*) were generated by CRISPR–Cas9 technique. Guide RNAs precisely targeting two sites of the gene were designed by using CHOPCHOP web tool (https://chopchop.rc.fas.harvard.edu/dev/) (Fig. S2A, B). The cas9-overexpressing plasmid was introduced in the parasites by electroporation at 450 volts (V) by using BIO-RAD GenePulsar X-cell with capacitance and resistance adjusted to 500 micro-Faraday (µF) and 100 Ohms (Ω), respectively. In the cas9-overexpressing parasites, the guide RNA-bearing plasmids were introduced by the same method. The cells were subjected to clonal selection by culturing them in M199 agar media in the presence of 50 µg·ml^−1^ hygromycin and 250 µg·ml^−1^ G418. The downregulation of the target gene in the selected colony was confirmed by RT-PCR analysis of the Atg8 transcript (Fig. S2C). Reintroduction of Atg8 in ∆*Atg8* cells was done via codon optimization to alter the target sites for gRNA recognition (Fig. S3B). CLUSTALW alignment showing a deleted portion of the *Atg8* gene in *Atg8* mutant cells is shown in Fig. S2D.

### Electron microscopy

Scanning electron microscopy (SEM) was used for morphological analysis of promastigotes and axenic amastigotes. Samples were fixed by using 2.5% glutaraldehyde in 0.1 M sodium cacodylate buffer overnight at 4 °C and postfixed in 1% osmium tetroxide. Thereafter, they were washed with sodium cacodylate buffer (pH 7.3), adhered on poly-L-lysine-coated coverslips, and dehydrated in an ascending series of ethanol. The samples were critical-point dried in CO_2_, coated with gold, and observed in a ZEISS EVO LS10 scanning electron microscope (Zeiss, Jena, Germany).

Transmission electron microscopy was used for ultrastructural analysis. After washing with 1× PBS, samples were fixed by using 2.5% glutaraldehyde in 0.1 M sodium cacodylate buffer (pH 7.3) overnight at 4 °C and postfixed in 1% osmium tetroxide. The parasites were dehydrated in an acetone series and embedded in araldite resin for 72 h at 60 °C. Staining of ultrathin sections of the embedded resin was done by using 5% uranyl acetate and lead citrate. Finally, the sections were examined in a JEOL JEM-2100 F/HR transmission electron microscope (Jeol, Tokyo, Japan).

### Infection

For infection experiments in vitro, THP1 cells (5 × 10^5^ cells ml^−1^) were differentiated into macrophages by using 50 ng·ml^−1^ Phorbol-12-myristate-13-acetate (PMA). After 72 h, the fully differentiated macrophages were incubated with stationary-phase *L. donovani* promastigotes at a ratio of 10 parasites/macrophage in serum-free medium for 3 h. Subsequently, the macrophages were washed several times with the same medium to remove non-phagocytosed promastigotes. The infected cells were maintained in RPMI supplemented with 10% FCS for varied time periods at 37 °C with 5% CO_2_ and air. For analyzing parasite Atg8 protein expression levels during differentiation, cell lysates were prepared from amastigotes isolated from infected macrophages. The infected macrophages were washed with 1× PBS to get rid of any extracellular parasite in the supernatant followed by treatment with 10 mM glucose in 1× PBS and lysed by passing through a 25-gauge syringe. The cellular and nuclear debris of macrophages were separated by centrifugation at 130 × *g* for 10 min. The supernatant was passed through a 3-µm filter obtained from Sigma-Aldrich (St. Louis, MO). Amastigotes were collected by centrifugation of the filtrate at 1800 × *g* for 10 min. Centrifugation was carried out by using Centrifuge 5810R (Eppendorf, Hamburg, Germany). Whole-cell lysates of the recovered amastigotes were then prepared as mentioned earlier under the “Western blots” section.

For in vivo experiments, separate batches of C57BL/6 mice were randomly selected for specified treatment, one batch was infected with wild type and the other with *Atg8* mutant parasites. A third set was used as uninfected control. The mice were infected through intracardiac route with 50 µl of 0.1% NaOH solution containing 10^7^ parasites followed by intraperitonial injection of an inoculum containing 10^8^ parasites at an interval of 1 week. The intraperitonial injections were repeated twice. After 30 days, the animals were humanely euthanized, the spleen size and weight were measured, and spleen smears were made on microscopy slides. The slides were stained with Giemsa dye and analyzed for the presence of isolated parasites and infected macrophages by light microscopy under oil immersion (1000×). Data obtained from all the animals were analyzed. The parasite burden was calculated in terms of Leishman–Donovan units (LDU), LDU = number of parasites per 500 nuclei × weight of the organ. The parasite counting was performed by a person in the laboratory blinded for the experimental conditions, other than the person performing the experiment.

### Statistical analysis

All experiments were independently repeated at least three times and represented as data ± SEM. To evaluate statistical difference and significance, an unpaired, two-tailed student’s *t* test was performed. For all experiments a *p* value of <0.05 was considered statistically significant. For comparing parasite burden and mice data between different test groups, where information about a Gaussian distribution could not be conclusively derived, Mann–Whitney test was performed. Wilcoxon signed-rank test was applied to analyze the data from the paired samples. The statistical analysis was performed by using GraphPad Prism, version 5.0 (GraphPad, San Diego, CA, USA).

## Supplementary information

Supplementary figure legends

Supplementary fig 1

Supplementary fig 2

Supplementary fig 3

Supplementary fig 4

Supplementary fig 5

Supplementary fig 6

Supplementary fig 7
