# Supplementary material for: Leishmania donovani parasite requires Atg8 protein for infectivity and survival under stress
Source: Cell Death Dis. 2019 Oct 24;10(11):808. doi: 10.1038/s41419-019-2038-7 (PMC6813314; doi:10.1038/s41419-019-2038-7)
Supplement: Supplementary file 1 — Supplementary figure legends [file 41419_2019_2038_MOESM1_ESM.pdf]

DECLARATION OF CONTRIBUTIONS TO ARTICLE

ADMC

Manuscript Number:	Journal Name:	
CDDIS-19-1816RR	*Cell Death & Disease*	(the 'Journal')

Proposed Title of the Contribution:

Leishmania donovani parasite requires Atg8 protein for infectivity and survival under stress (the 'Contribution')

Author(s):

Sagnik Giri and Chandrima Shaha (the 'Authors')

For all *CDDis* articles, each person named as an author in the published version must be able to show he or she has contributed substantially to the article.

Authorship credit should be based on 1) substantial contributions to conception and design, acquisition of data, or analysis and interpretation of data; 2) drafting the article or revising it critically for important intellectual content; and 3) final approval of the version to be published. Authors should meet conditions 1, 2 and 3.

Any person who cannot be shown to have made a substantial contribution to the article cannot be listed as an author in the final version. The name of any person who is deemed to have made a minor contribution can, however, appear in the Acknowledgments section of the article.

Please complete the table below to indicate the contributions of all named authors to the manuscript.

Author Full Name:	Specification of Contribution to the Manuscript:
Sagnik Giri	Designing experiments, performing experiments, writing manuscript
Chandrima Shaha	Designing experiments, supplying materials, writing manuscript

Please complete the table below to indicate the contributions of all named authors to the figures.

Figure 1:
Sagnik Giri and Chandrima Shaha

Figure 2:
Sagnik Giri and Chandrima Shaha

Figure 3:
Sagnik Giri and Chandrima Shaha

Figure 4:
Sagnik Giri and Chandrima Shaha

Figure 5:
Sagnik Giri and Chandrima Shaha

Figure 6:
Sagnik Giri and Chandrima Shaha

Signed for and on behalf of the Author(s):	Print Name:	Date:
Chandrima Shaha	Chandrima Shaha	27 September, 2019
