# Supplementary figures and images for: Leishmania donovani parasite requires Atg8 protein for infectivity and survival under stress
Source: Cell Death Dis. 2019 Oct 24;10(11):808. doi: 10.1038/s41419-019-2038-7 (PMC6813314; doi:10.1038/s41419-019-2038-7)

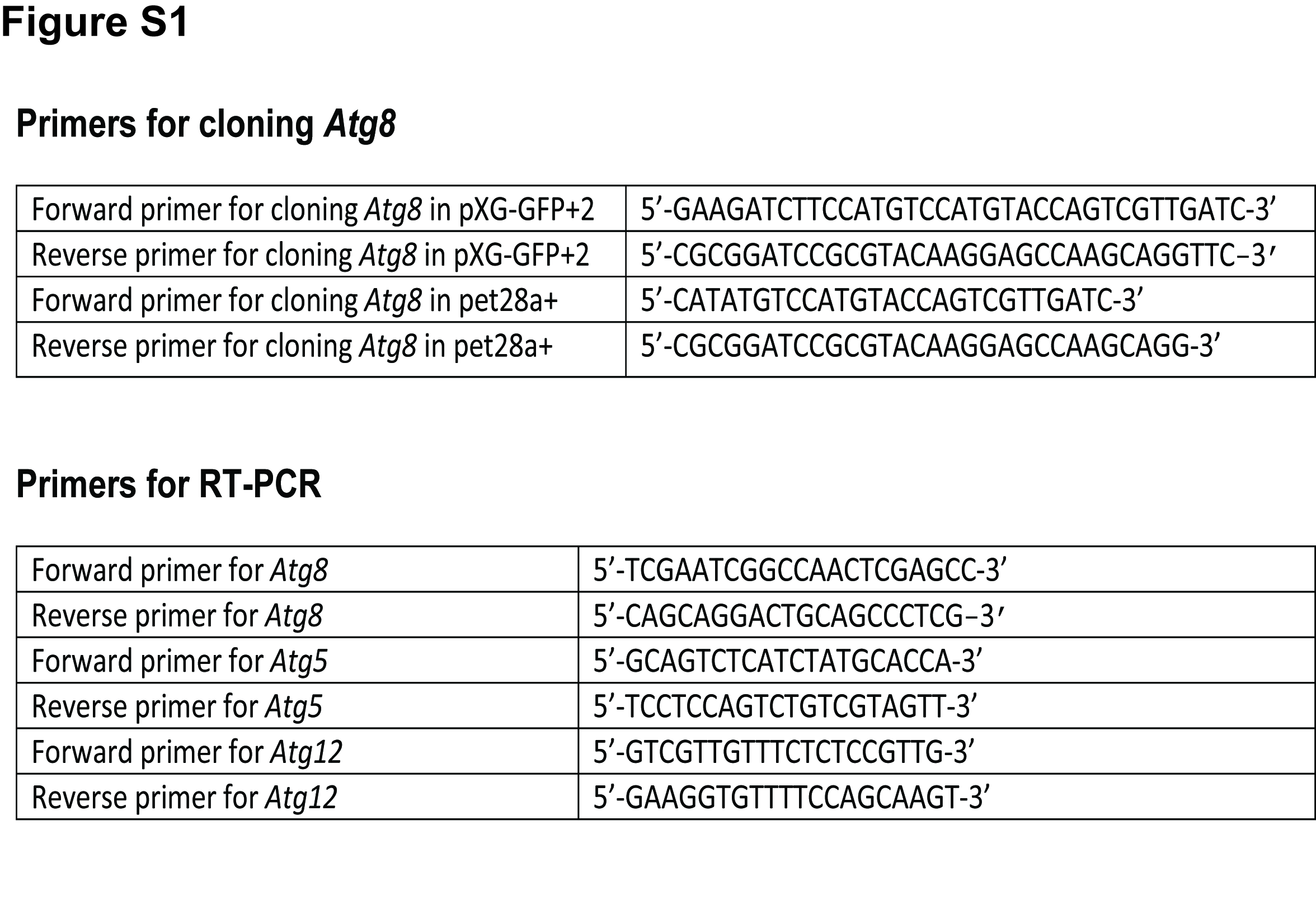

Supplement: Supplementary file 2 — Supplementary fig 1 [file 41419_2019_2038_MOESM2_ESM.tif]

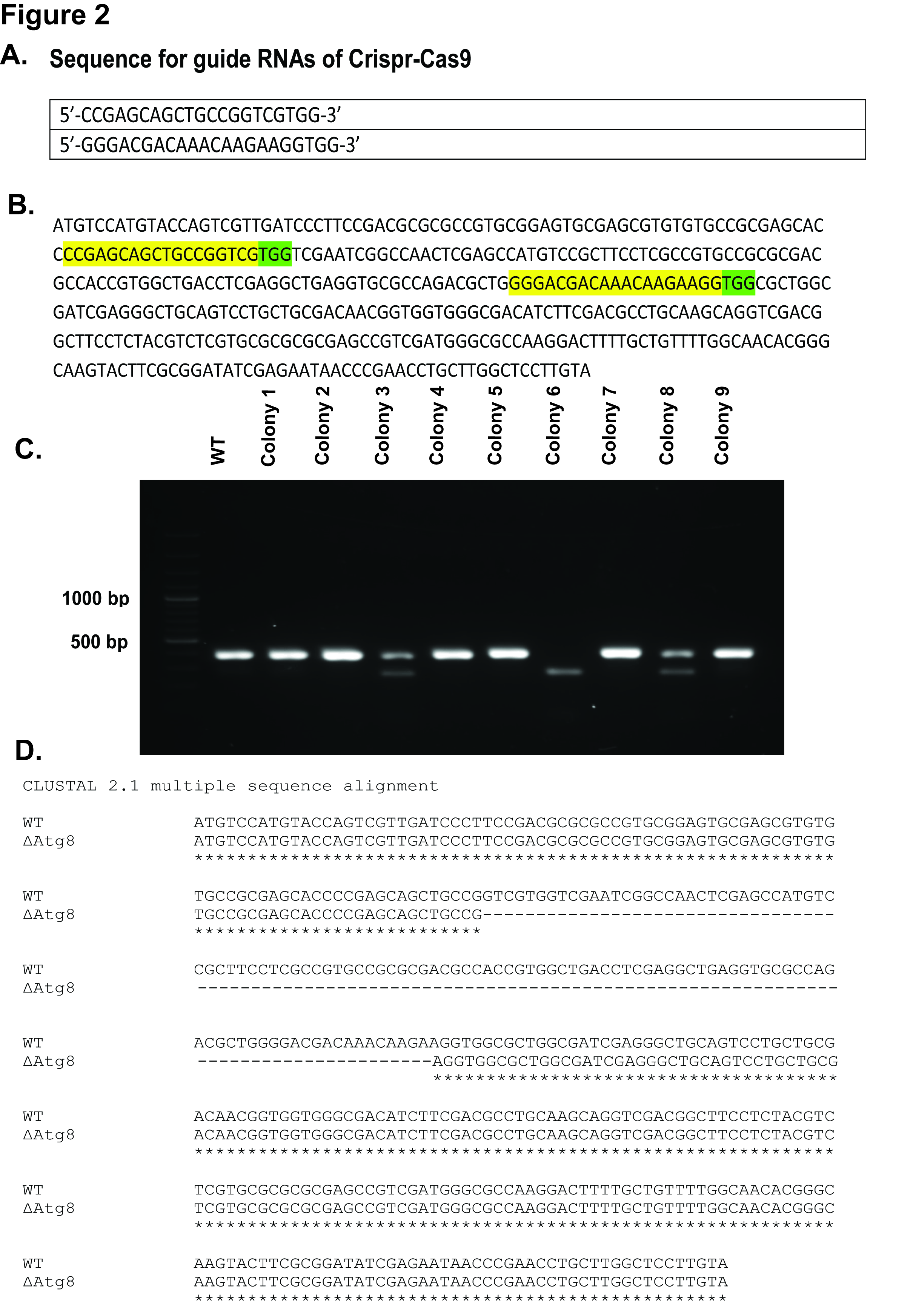

Supplement: Supplementary file 3 — Supplementary fig 2 [file 41419_2019_2038_MOESM3_ESM.tif]

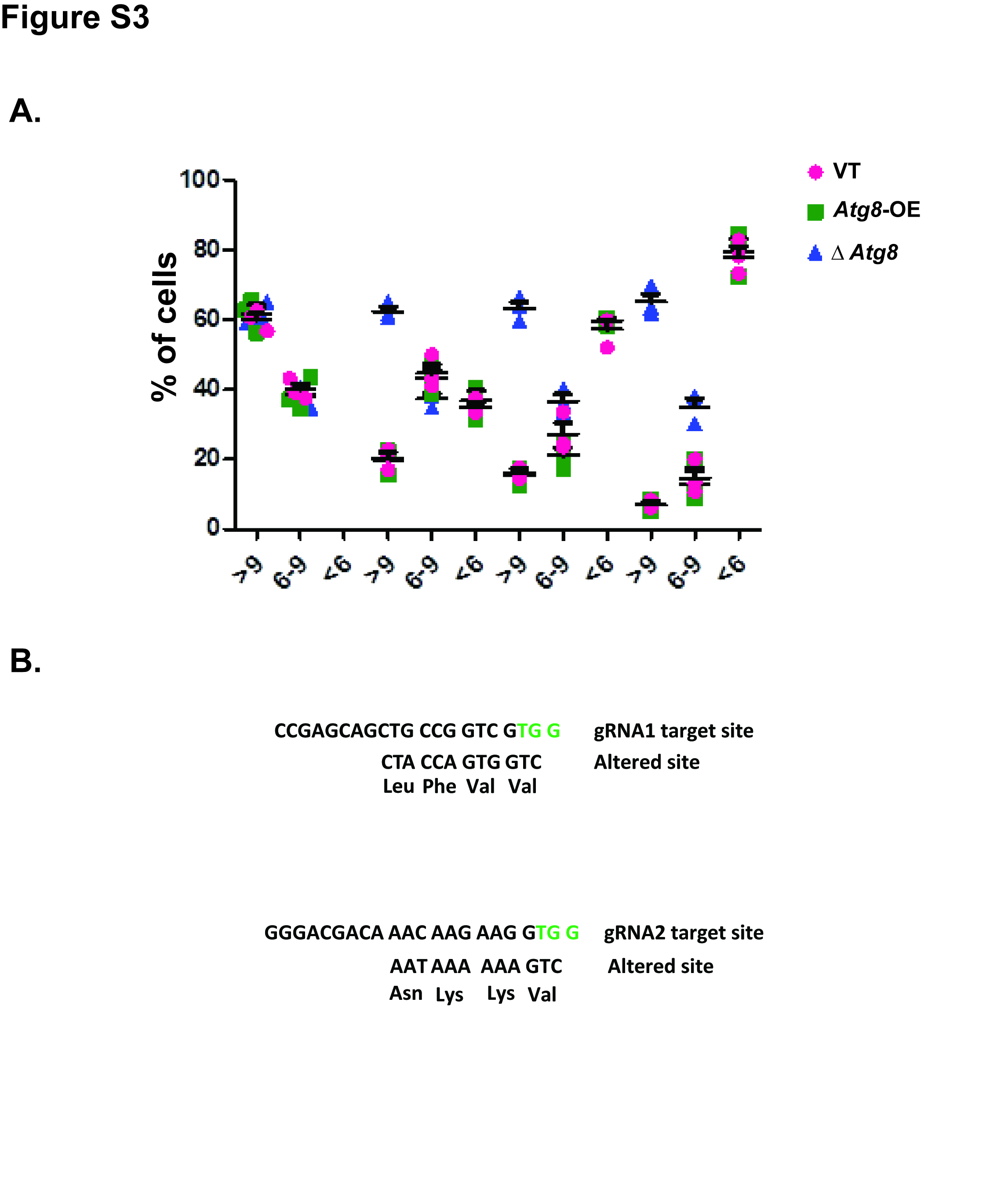

Supplement: Supplementary file 4 — Supplementary fig 3 [file 41419_2019_2038_MOESM4_ESM.tif]

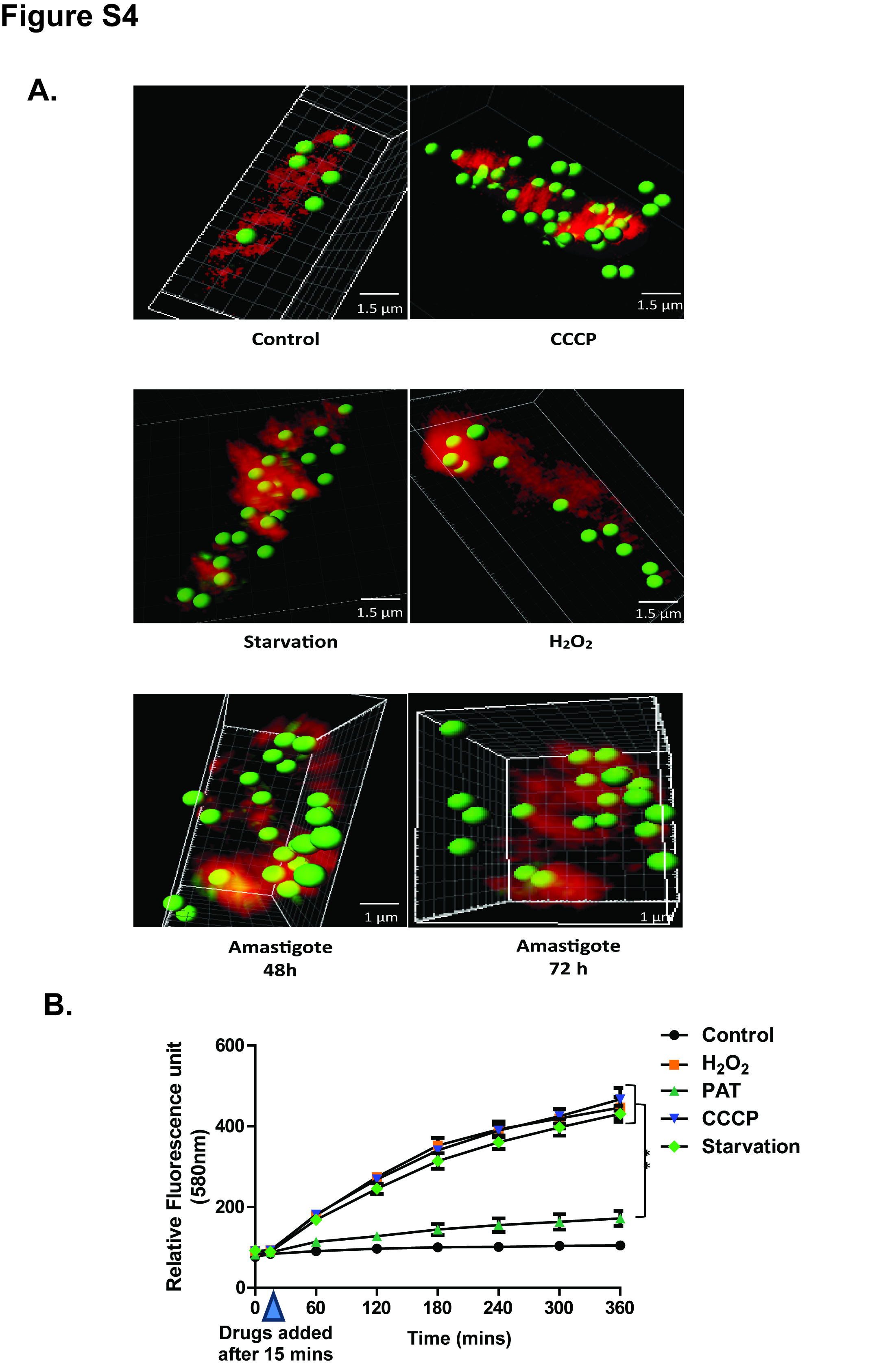

Supplement: Supplementary file 5 — Supplementary fig 4 [file 41419_2019_2038_MOESM5_ESM.tif]

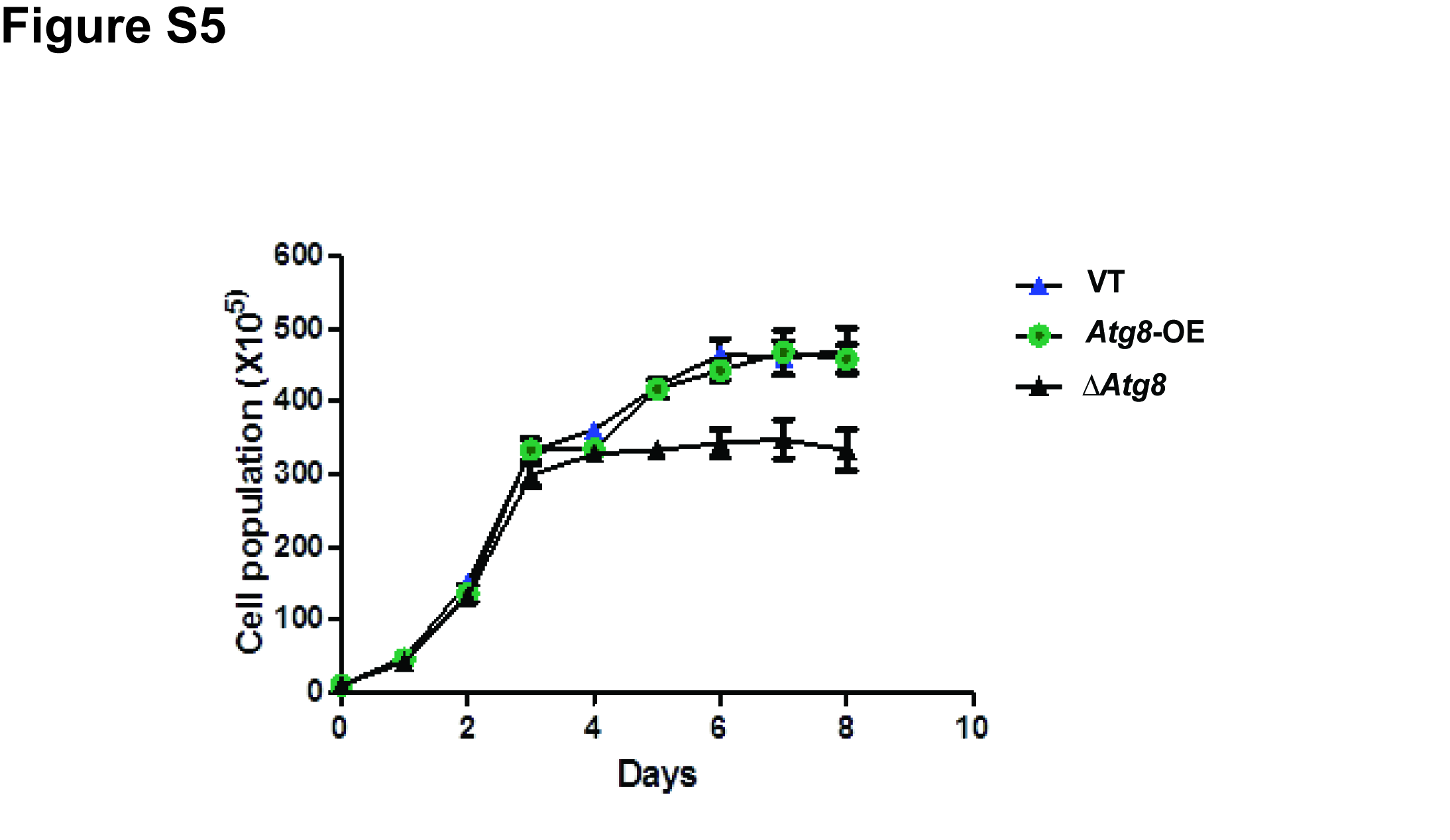

Supplement: Supplementary file 6 — Supplementary fig 5 [file 41419_2019_2038_MOESM6_ESM.tif]

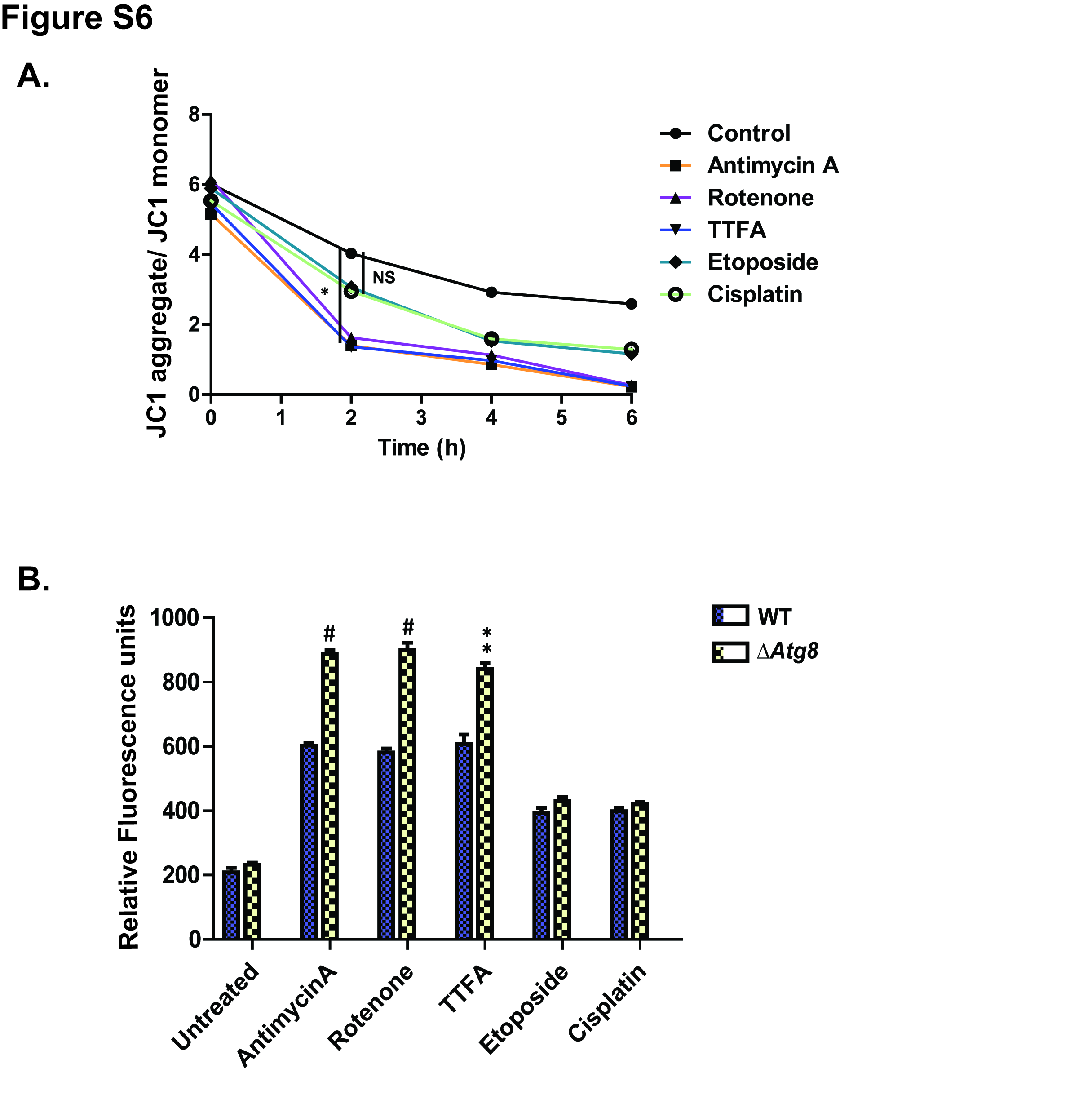

Supplement: Supplementary file 7 — Supplementary fig 6 [file 41419_2019_2038_MOESM7_ESM.tif]

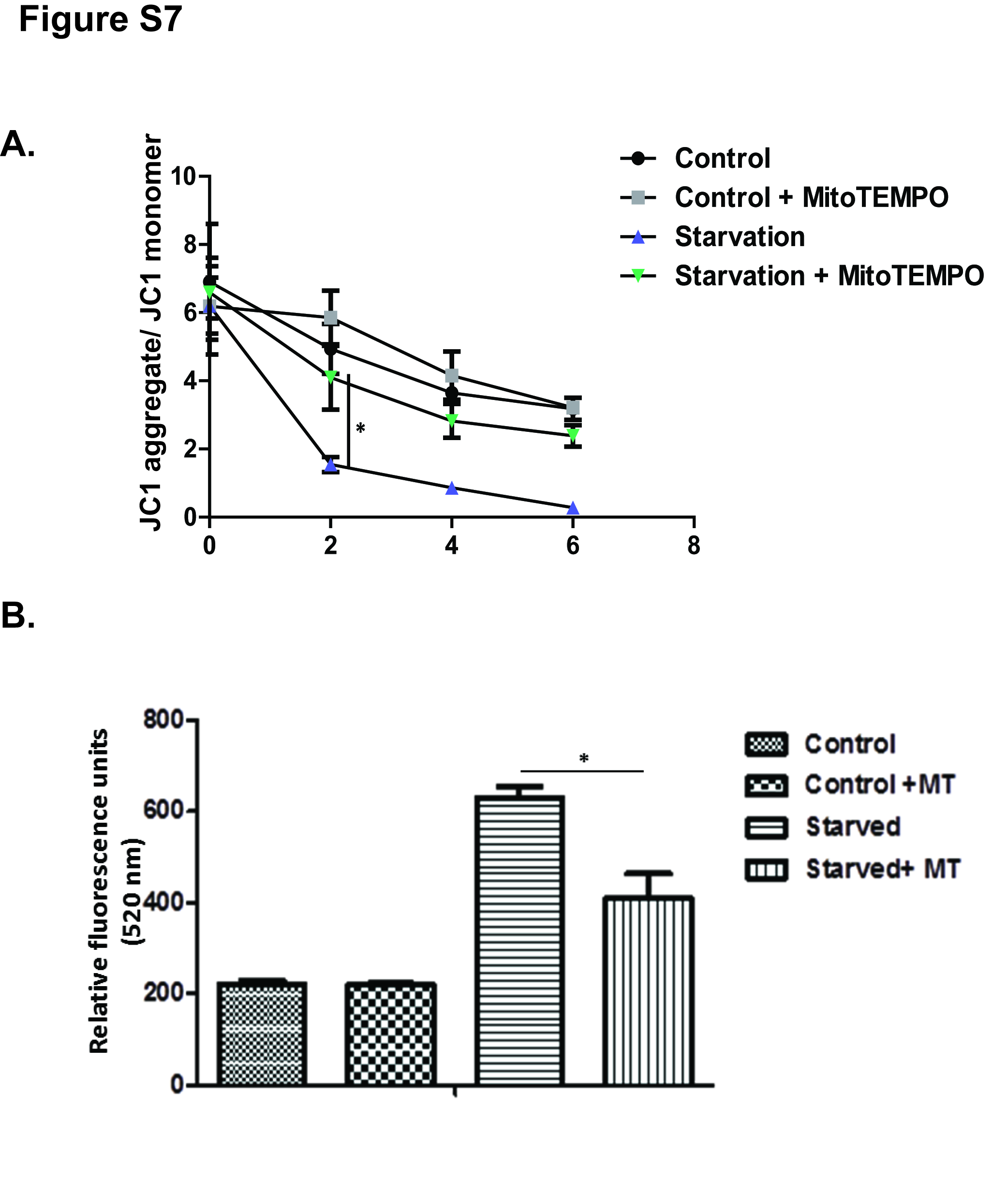

Supplement: Supplementary file 8 — Supplementary fig 7 [file 41419_2019_2038_MOESM8_ESM.tif]
